# Efficient, Capsule‐Free Nymphal Ixodid Tick Placement for Site‐Specific Attachment to Assess Anti‐Tick Host Behavior and Immune Responses

**DOI:** 10.1002/cpz1.70477

**Published:** 2026-09-23

**Authors:** Ronja A. Frigard, Serena Doh, Charles S. Grugan, Yanling Liu, Sara I. Mirbagheri, Luana A. Rogerio, Eva Iniguez, Tiago D. Serafim, Aline da Silva Moreira, Jack Collins, Daniel E. Sonenshine, Jesus G. Valenzuela, Johannes S. P. Doehl

**Affiliations:** ^1^ Vector Molecular Biology Section, Laboratory of Malaria and Vector Research, National Institute of Allergy and Infectious Diseases National Institutes of Health Rockville Maryland; ^2^ Integrated Data Sciences Section, Research Technologies Branch, Division of Intramural Research National Institute of Allergy and Infectious Diseases Bethesda Maryland; ^3^ Laboratory of Malaria and Vector Research, National Institute of Allergy and Infectious Diseases National Institutes of Health Bethesda Maryland

**Keywords:** *Amblyomma americanum*, *Dermacentor variabilis*, *Ixodes scapularis*, placement, *Rhipicephalus sanguineus*

## Abstract

Ixodid ticks are among the most widespread and permissive groups of arthropod disease vectors in the world. They transmit various viral, bacterial, protozoan, and even nematode pathogens infective for humans and animals. Ticks are essential to studying pathogen transmission and infection in a new host; however, exposing in vivo models to ticks is very challenging. Most current tick placement protocols rely on capsules to increase attachment rates at a specific location. However, capsules interfere with hosts’ ability to engage with ticks by grooming/scratching. Conversely, available capsule‐free tick infestation protocols, including free‐roaming protocols, suffer from low and highly variable tick attachment efficacy. Here, we describe a highly efficient capsule‐free nymphal tick placement protocol with tick attachment rates of ≥85% across various tick species and genera, offering the generation of highly reproducible data. This article also offers a set of protocols to the user primarily geared toward the study of itch‐induced tick removal but are adaptable to other study settings. Published 2026. This article is a U.S. Government work and is in the public domain in the USA. Current Protocols published by Wiley Periodicals LLC.

**Basic Protocol 1**: Capsule‐free, highly efficient multi‐species nymphal tick placement for site‐specific attachment

**Basic Protocol 2**: Assessing efficacy of itch‐induced tick removal by quantifying loss of ticks over time

**Basic Protocol 3**: Assessment of health/ability of partially fed nymphal ticks to feed determined by scutal index

**Basic Protocol 4**: Scratch bout quantification in guinea pigs

**Support Protocol**: Development of a semi‐supervised deep learning pipeline for automated detection and validation of scratching behavior

**Basic Protocol 5**: Assessment of local skin inflammation due to immunity to tick bites using laser speckle contrast imaging

## INTRODUCTION

Ixodid ticks are the most widespread and permissive group of arthropod vectors of disease in the world (Beard et al., 2021; R. J. Eisen & Paddock, 2021; Madison‐Antenucci et al., 2020; Sonenshine & Roe, 2013). They transmit various viral, bacterial, protozoan, and even nematode pathogens infective for humans and animals, causing dozens of different infectious diseases, including Lyme disease, tick‐borne encephalitis, and Babesiosis (Brites‐Neto et al., 2015; Nicholson et al., 2019). Most tick‐borne diseases could be prevented by early tick removal, as most tick‐borne pathogens require hours, if not days, post‐tick bite to be transmitted (L. Eisen, 2018). We recently described an early neuro‐immunological anti‐tick response that we termed itch‐induced tick removal (IITR) (Doehl et al., 2026). IITR is rapidly acquired within 5 days post initial tick exposure and becomes evident within 3 to 4 hr post subsequent tick bites. In sensitized guinea pigs, IITR results in the rapid removal of biting ticks prior to the transmission of most tick‐borne pathogens. Thus, IITR may be exploited as a tick‐borne pathogen transmission intervention; a single treatment to prevent multiple diseases.

To study IITR, guinea pigs must be regularly exposed to ticks. We utilize nymphal ticks, which are more visible to the observer than larvae, but small enough to place at least 15 well‐spaced ticks on the guinea pig's head. Their slower motility also makes it easier to handle them in larger numbers in a capsule‐free setup than adult ticks. Most current tick placement protocols use capsules to confine ticks to increase attachment rates at a specific location (Narasimhan et al., 2024). However, capsules interfere with animals’ ability to scratch ticks off. Conversely, available capsule‐free, also known as free‐roaming, tick infestation protocols suffer from potentially low and highly variable attachment efficacy (Kryda et al., 2025; Otaki et al., 2018). This is problematic for data integrity. For example, a host with multiple ticks represents a potential source of data variability even if the unit measured is the tick. In the case of tick removal on a host with many attached ticks, each tick bears less statistical weight than in a case where there are only a few ticks attached. In other words, in the case of a host with 20 attached ticks, each removed tick only has a 5% weight on the total ratio of removed ticks, while in the case of only 5 attached ticks, every removed tick has a 20% weight. Thus, to balance the data weight of each tick, ideally equal numbers of ticks are attached per host. It is noteworthy that, to our current knowledge, IITR intensity is equivalent whether 5 or 20 nymphal ticks were placed on a guinea pig. Also, tick attachment at random body locations makes it difficult to observe ticks during feeding, as they may escape observation by the researcher. These problems create significant reproducibility issues. Our new capsule‐free protocol addresses these issues and controls for attachment efficacy and location by promoting biting (see Basic Protocol 1 for details).

IITR activity is directly observed by counting ticks that remain attached at specific time points post‐placement. The data are collected as time‐to‐event, also known as survival, datasets and can be graphically represented by a Kaplan–Meier graph. The data are best analyzed by a Cox proportional hazards model for the data hierarchy created by having multiple ticks attached per guinea pig (see Basic Protocol 2 for details).

Assessment of tick health has traditionally been done by assessing tick engorgement weight, egg mass weight, and egg hatching (Huercha et al., 2020). However, these latter metrics are specific to adult ticks and not to larvae or nymphs, while the former becomes difficult to measure for larvae and nymphs due to their low weight even after full engorgement. To circumvent these limitations, we adapted the scutal index described by Meiners et al. (2006) as a measure of feeding success. This approach does not require completion of engorgement and is tick‐stage independent. The protocol only requires sufficient magnification on your stereomicroscope, resolution on the camera, and software to make measurements (see Basic Protocol 3 for details).

Measuring itch activity directly in vivo is very complex as it is a neurological response. However, the mechanical response to itch can be easily observed externally in a live animal. Scratching is frequently used in the peer‐reviewed literature as a surrogate measure for itch and itch intensity (Erickson & Kim, 2019; Felix & Shuster, 1975). Data collection is routinely done in mice by simply looking into the cage for a given duration and recording the scratch events. This does not work reliably with guinea pigs. Their natural response to a threat is to freeze, which includes the suppression of scratching. Recording guinea pigs in a room free of human activity is a reliable way to assess scratching behavior. Guinea pigs are often referred to as being crepuscular (most active during twilight hours). In fact, guinea pigs do not follow a typical sleeping cycle, but are active around the clock, sleeping intermittently in short bursts throughout a 24‐hr cycle (Lee et al., 2014). Therefore, scratch behavior observations at nighttime are equally valid as daytime observations. To manage the analysis of such long recordings, we developed a recording analysis app written in Python and based on a deep learning neural network, which reliably identifies scratching events in the recordings and is freely available for download (https://github.com/IDSS‐NIAID/tick‐scratch‐detection; see Basic Protocol 4 and Support Protocol for details).

Non‐invasive in vivo techniques are highly significant as they permit repeated and varied observations in the same individual, reducing data variability. We employ laser speckle contrast imaging to observe inflammation and cellular infiltrate into tick bite sites. This non‐invasive imaging technology has been in clinical use since the late 1990s and has been primarily used to observe blood flow (Heeman et al., 2019). However, the principle of laser speckle contrast imaging is based on light refraction, which is more variable for a moving object compared to a stationary one. Immune cells recruited into the skin are not stationary but actively move through the dermis at the tick bite site. In doing so, their light refraction patterns are much more variable compared to the relatively stationary cells of the skin. This difference is detectable by laser speckle contrast imaging, allowing a non‐invasive assessment of cellular infiltration into tick bite sites (see Basic Protocol 5 for details).

## STRATEGIC PLANNING

We recommend working at a minimum in pairs. Two people are required for the administration of the anesthesia in guinea pigs. We also recommend handling no more than 9 guinea pigs at a time when working in pairs. Anesthetize up to 3 guinea pigs at a time or do the anesthesia sequentially by applying anesthesia to the guinea pigs in 7‐ to 10‐min intervals.

To upscale the protocol, you can either stagger the nymphal tick exposures over several days, or you can have more hands available to conduct the work. When more people are available, they can be assigned to specific tasks, which can increase productivity. For example, with three people, one can do the tick placement while another does the dapping and monitoring of the placed ticks. The third can monitor the guinea pigs, do the shaving, and manage the anesthesia.

While it is possible to undertake nymphal tick infestations weekly on the same guinea pigs, scratching may damage their skin during later nymphal tick exposures, leaving the placement side covered in scabs. This may require resting guinea pigs for 1 to 2 weeks to let the skin heal, delaying experimental procedures. However, resting the guinea pigs for an additional week will not affect the results. When working with *Amblyomma americanum* and *Rhipicephalus sanguineus*, guinea pigs tend to develop many scabs by the third nymphal tick exposure due to rapid onset of skin cavitation at the bite site (Anderson et al., 2017). Skin cavitation is a consequence of an aggressive host immune response against the ticks, hallmarked by large cellular influx into the bite site.

We realize that the number of sub‐step points and step annotations in the protocols are considerable and may be distracting to some users trying to follow the protocol. However, the main points of each protocol are contained within each numbered step of each protocol. The sub‐step points and annotations merely contain additional tips, explanations, or optional modifications to the protocols. To improve the workflow, we recommend highlighting the numbered steps along with any modifiers in the sub‐step points that are needed to execute the protocol in the desired form.

*CAUTION*: All placements of uninfected nymphal ticks should be conducted in a contained environment to prevent unobserved loss of nymphs. We use a biological safety hood with a turned‐off fan. The draft may disperse nymphs suddenly while transferring them from a vial to a guinea pig. We also recommend laying out white absorbent material like blue pads on which to place the shaved guinea pigs. This will absorb urine, which guinea pigs commonly lose during general anesthesia, and it will make spotting dropped nymphs easier.

*CAUTION*: It is recommended to place animals on heating mats at 37°C during tick exposure. The heating mats should be placed under the blue pads. This will help them maintain their body temperature during anesthesia, preventing hypothermia. This improves animal recovery from anesthesia and tick attachment.

*CAUTION*: Working with infected nymphal ticks should always be conducted in accordance with local biosafety level rules. All infected nymphs must be accounted for after placement. An infected nymph is fully capable of transmitting tick‐borne pathogens and, therefore, represents a health risk to the investigator, his/her coworkers, and other staff.

*CAUTION*: When using Elizabethan collars on guinea pigs in the containment cages post tick placement, place guinea pig chow in small metal pet food trays. J‐feeders should be avoided as Elizabethan collars may catch on the corners of the J‐feeder, potentially causing injury to the guinea pig. The placement of the Elizabethan collar must not choke the guinea pig. Therefore, there must be a one‐finger‐wide space between neck and collar. This is best assessed while the guinea pig is anesthetized, and the neck is relaxed.

*CAUTION*: Depilatory creams contain harsh chemicals, like potassium thioglycolates and calcium hydroxide. Prolonged exposure to these creams can cause chemical burns to the skin and kill attached ticks. Therefore, it is crucial to reduce depilatory cream exposure to the minimum necessary and remove any residual cream after the depilation process is complete.

*NOTE*: All protocols involving animals must be reviewed and approved by the appropriate Animal Care and Use Committee and must follow regulations for the care and use of laboratory animals.

## Basic Protocol 1 CAPSULE‐FREE, HIGHLY EFFICIENT MULTI‐SPECIES NYMPHAL TICK PLACEMENT FOR SITE‐SPECIFIC ATTACHMENT

**Figure 1:**
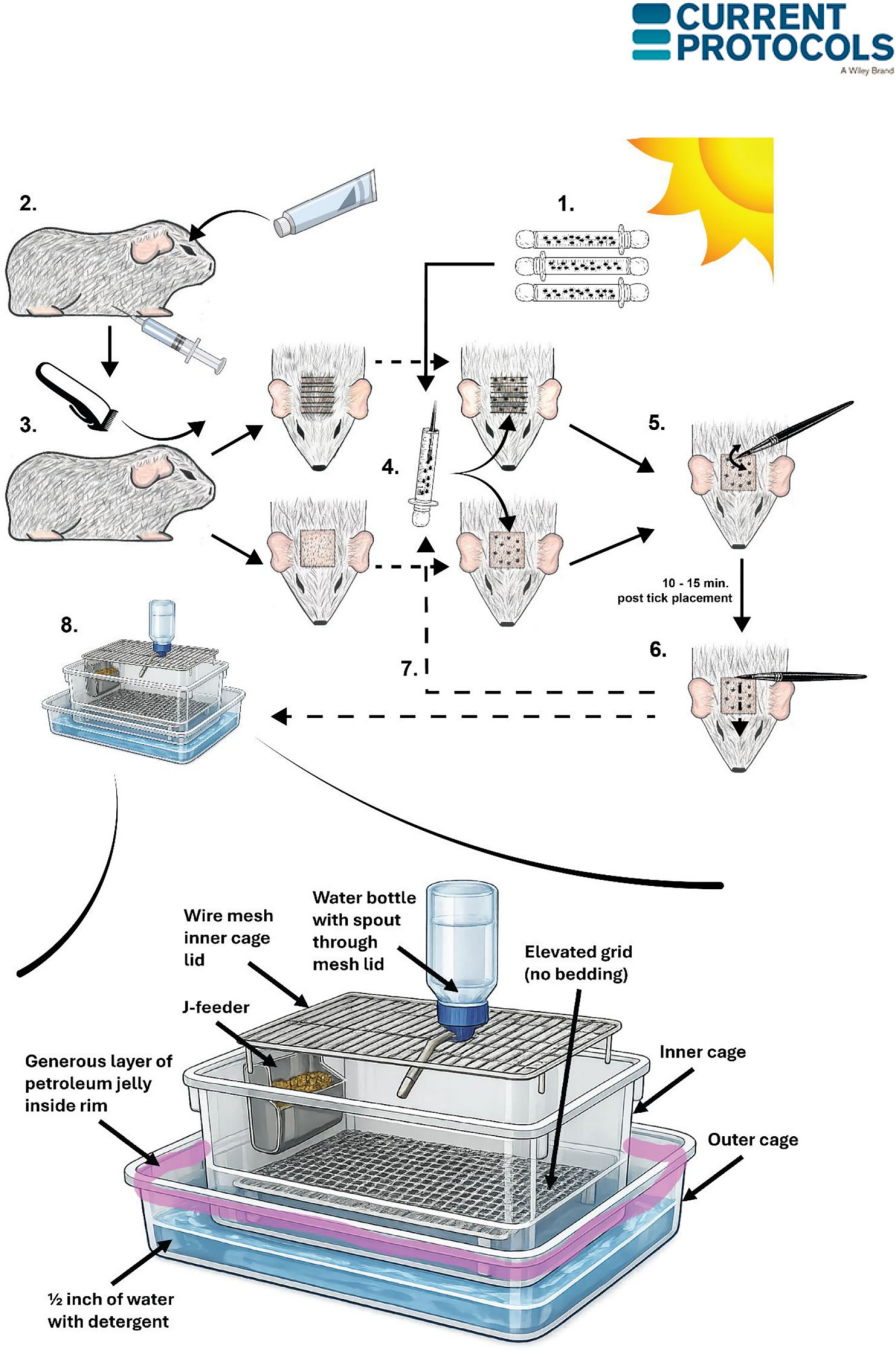
Schematic representation of the tick placement protocol. (1) Expose ticks to sunlight some days before use. (2) Anesthetize guinea pigs and protect eyes with artificial tear ointment. (3) Shave the tick‐placement area according to tick species. Leave a stubble. (4) Place ticks on the shaven area. (5) Dab ticks gently on their rear to promote burrowing into the fur stubble. Continue to intermittently dab burrowed‐in ticks for 10 to 15 min. (6) 10 to 15 min post tick placement, brush against the grain to displace unattached ticks. (7) Replace unattached ticks with fresh ones by repeating steps 4 and 5. (8) Once sufficient ticks seem attached, recover guinea pigs, and return them to triple‐containment cages for isolated housing. Elements of the triple‐containment cage are detailed in the expanded figure. Elements of the workflow were produced by Sara I. Mirbagheri or supplied by the BioART department/NIH.

**Figure 2:**
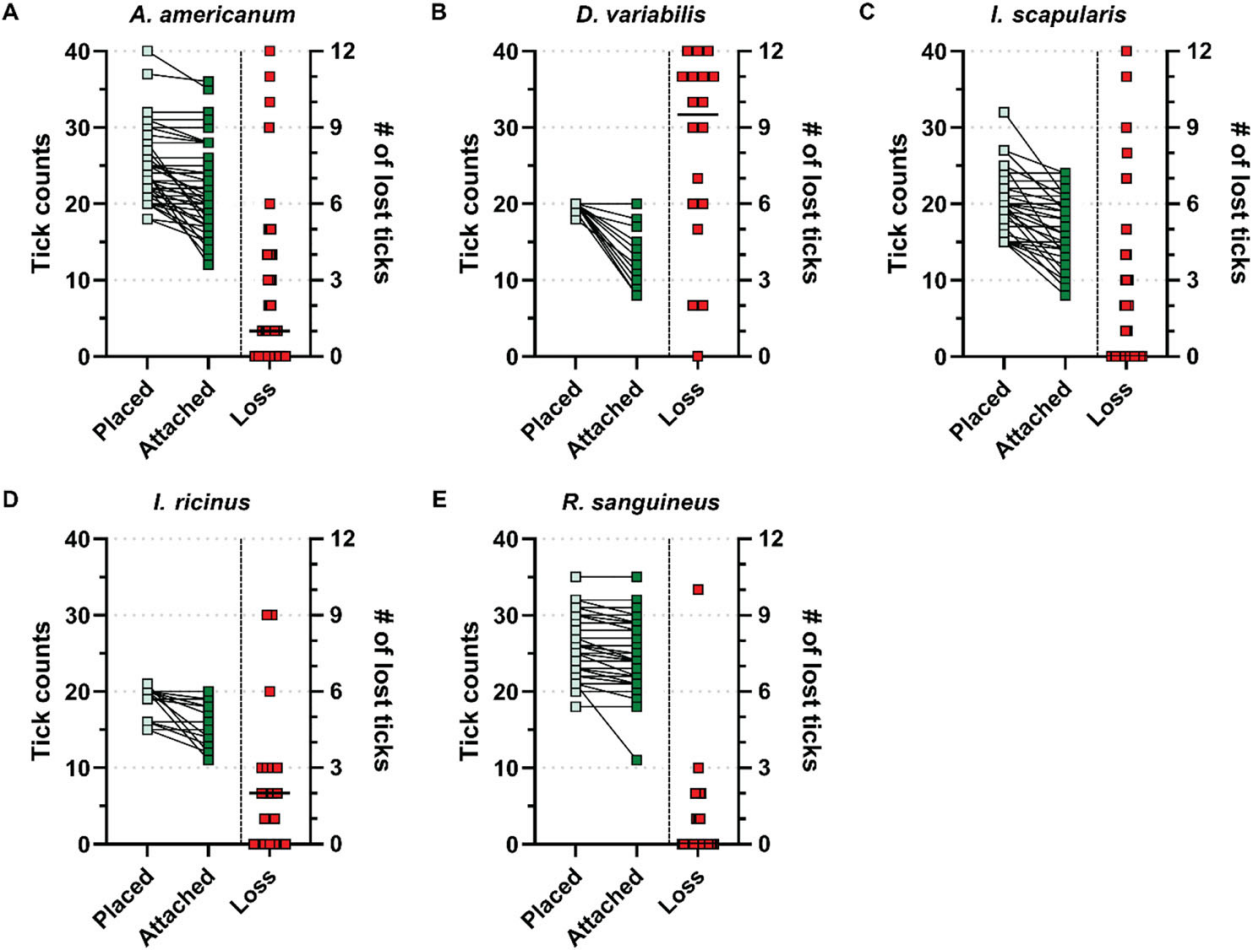
Paired graphs showing the number of placed ticks (light green) and the number that were found attached (dark green) by 3 hr post placement for guinea pigs exposed to (**A**) *A. americanum*, (**B**) *D. variabilis*, (**C**) *I. scapularis*, (**D**) *I. ricinus*, and (**E**) *R. sanguineus* nymphs. The scatter plot data in red show how many nymphal ticks per guinea pig had dropped or remained unattached by 3 hr post tick placement.

This protocol describes the placement of various nymphal tick species and was developed using guinea pigs (Fig. 1). We have used this protocol successfully with *Amblyomma americanum*, *Ixodes scapularis*, *Ixodes ricinus*, and *Rhipicephalus sanguineus* nymphs. The protocol is simple, efficient, reproducible, and capsule‐free (≥85% attachment success consistently; Fig. 2). The protocol is also applicable to *Dermacentor variabilis* but less efficient due to the slow attachment of these nymphs. There are minor alterations to the nymphal tick placement protocol, depending on which ixodid tick species you are working with. The alterations will be pointed out in the relevant places. If needed, removal of ticks by grooming and induced scratching can be inhibited by placement of an Elizabethan collar, while ticks are placed on the guinea pig's head. Inclusion of this alternative is indicated below. Making minor adjustments, we have also used this protocol successfully with mice and *I. scapularis* nymphs. Other tick species have not been tested with mice so far. The adjustments are indicated in the relevant places in the protocol below. The aim of this protocol is the reproducible attachment of a desired number of nymphal ticks per guinea pig without excessive wastage of ticks and off‐target‐site biting. If you do not have a access to a tick colony at your institute, or through a collaborator, in many countries there are institutions that offer all tick life‐stages for purchase. For example, within the USA, you can purchase various locally‐occurring tick species from the the Centralized Tick Rearing Facility, Department of Entomology and Plant Pathology, Oklahoma State University, Stillwater, Oklahoma, like we did.

### Materials

Nymphal ixodid ticks
Albino Hartley guinea pigs (*Cavia porcellus*; Charles River, strain no. 051)
Petroleum jelly (e.g., Vaseline)
Anesthesia (see recipe)
Lubricant eye ointment (e.g., “Systane” from Alcon)
Isoflurane (e.g., “Forane” from Baxter)

Desiccator cabinet (e.g., SP Bel‐Art Secador polystyrene mini gas‐purge desiccator cabinet; 0.3 cubic ft from Novatech, cat. no. SPB‐F42075‐1002)
Ziploc bags
Kitchen sponges
Access to sunlight, e.g., a window
1‐ml syringes (e.g., from BD)
27G × 0.5 in. (13 mm) sterile needles (e.g., “PrecisionGlide” from BD)
Electric contour clippers (e.g., BravMini+ from Wahl)
Medical blue pads
Heating mats
Sticky tape (preferably double‐sided)
Elizabethan collars (Rat from Kent Scientific, cat. no. EC404VL‐10)
Fine paint brushes
Fine‐tipped, curved tweezers (e.g., Dumont #7 forceps from FST, cat. no. 11272‐50)
Scissors
Isoflurane vaporizer (e.g., from Vetequip or “SomnoFlo” from Kent Scientific)

Additional equipment for housing animals (see description in step 3)

#### Prepare nymphal ticks

There are various published protocols on nymphal tick housing. Some use plastic tubes sealed with vented membrane lids, cotton wool, or fine mesh. Cotton wool sealed cut‐off 3‐ml syringes are also used. For routine long‐term storage, plaster‐filled tubes may be preferred for their enhanced humidity and moisture regulation abilities (Sonenshine & Roe, 2013). For long‐term storage, tubes are placed in an incubator or insect chamber set to 20°C, high humidity, and a 16 hr light and 8 hr dark cycle. It is recommended to place tubes in Ziploc bags for additional tick containment and to preserve high humidity. The latter can be aided by adding moist paper towels or kitchen sponges to the Ziploc bag.

1 For tick placement, remove nymphal ticks from the holding incubator several days before the experiment. The tubes are double bagged in Ziploc bags containing pieces of moist paper towels or kitchen sponges for moisture.
2 We recommend placing the double‐bagged ticks at room temperature into direct sunlight (ideally morning sun) before use, but away from radiators.
  Different tick species have different requirements of minimum sunlight exposure before diapause is broken, and they are more likely to bite a host. During winter months, this time may need to be extended. For *A. americanum*, this step is indispensable.
*A. americanum*: A minimum of 7 days in direct sunlight
*D. variabilis*: A minimum of 5 days in direct sunlight
*I. scapularis*: A minimum of 4 days in direct sunlight
*I. ricinus*: A minimum of 4 days in direct sunlight
*R. sanguineus*: A minimum of 2 days in semidirect sunlight.

#### Prepare the guinea pigs

Albino Hartley guinea pigs (*Cavia porcellus*) were purchased from Charles River Laboratories, Wilmington, MA, and were communally housed in groups of up to three animals under static cage conditions. We have also tested this protocol on Strain 13 guinea pigs and found it to be equally valid.

3 Prepare triple‐containment cages for individual guinea pig housing post tick placement according to your specification (Fig. 1).
  a. Our containment setup consists of double caging. The outer cage will contain enough water with a bit of detergent to cover its bottom. The detergent breaks water surface tension and removes lipid layers from the tick surface, reducing the chances of a tick walking through the water. A generous layer of petroleum jelly (e.g., Vaseline) is applied on the inside of the outer cage pan rim. Nymphs get stuck in the jelly should they attempt to crawl through it. The inner cage pan contains an elevated wire mesh for urine and feces to drop below. No bedding is allowed as ticks will hide in the bedding. The inner cages are closed with a wire lid and maintained under static conditions. Guinea pig chow is placed in a J‐feeder or a small metal pet food tray. Water bottles are placed on top of the wire lid with spouts sticking through the wire lid.
  b. Alternatively, when using Elizabethan collars, avoid the use of J‐feeders as the Elizabethan collars may catch on the corners of the J‐feeder. Guinea pigs may also attempt to use the J‐feeder as leverage to force the Elizabethan collar off, which can cause injury to the animal. Thus, food must be placed in small metal pet food trays. Ideally, these will be wider at the bottom and narrower at the top, to prevent them from tipping over.
  c. For mice, triple‐containment cages are in a similar double‐cage setup. The same external cage with water moat and petroleum jelly should be used. The internal cage we use is from Lenderking and has four parts. The main cage is plastic, with holes in the floor to allow waste and ticks to drop through. The bottom of this cage is protected by a sealed tray that contains ticks and waste and allows the investigator to monitor for dropped ticks without opening the main cage. If gaps are observed between the main cage and bottom tray, particularly due to warping of the cages during autoclaving, petroleum jelly can be used to seal them. If rubber gaskets are available to seal the cages, they may also be used. The main cage is topped by a wire feeding rack with a bottle and chow, and a sealed, paper‐ventilated lid.
4 Weigh guinea pigs to determine anesthesia volume per guinea pig.
5 Anesthetize guinea pigs by intraperitoneal injection of a Ketamine (50 mg/kg) and Xylazine (5 mg/kg) anesthesia mixture (see Reagents and Solutions).
6 Once sedated, apply lubricant eye ointment and proceed to trim the hair on the crown of the guinea pig's head between its ears and its neck area with electric contour clippers. Ensure that a 1‐mm stubble remains (essential).
  We use two different trimming approaches depending on tick species:
  a. For *A. americanum*, instead of trimming the whole area, cut cross‐sectional ∼3‐mm wide trenches into the guinea pig's fur, while leaving ∼3 mm of untrimmed fur between trenches (Fig. 1). Within the trenches, only a 1‐mm stubble remains. This approach improves *A. americanum* nymphal attachment.
  b. For all other nymphal tick species tested, the whole target area can be trimmed down to a 1‐mm stubble. The stubble is essential as it allows the nymphs to burrow into the fur with immediate contact to the skin. Nymphal ticks do not like clean‐shaven skin as that precludes stimulation of dorsal tick sensilla, which promotes biting.
  c. Mice can be shaved similarly to guinea pigs using clippers. Care must be taken as mouse skin is very thin and fragile, and nipping the skin can cause bleeding and scabbing. Stubble is as helpful in mice as in guinea pigs, but in mice, tick placement can also be done by directly “inserting” the mouthparts into the thin skin.
7 Place guinea pigs on blue pads in a contained environment.
  We recommend placing heating mats at 37°C underneath the blue pads. This will help the guinea pigs to maintain their body temperature during anesthesia. This will also aid recovery from anesthesia and may improve tick attachment as it prevents hypothermia.

#### Tick placement

To avoid unobserved loss of nymphal ticks during placement, we recommend conducting the nymphal tick placement within a contained space like a containment hood. However, we recommend turning off the hood's fan to avoid nymphs from being blown away by the draft. We recommend laying out white absorbent material like blue pads on which to place the shaved guinea pigs. For one, this will absorb urine, which guinea pigs commonly lose during general anesthesia, and it will make spotting dropped nymphs easier. We also recommend placing a strip of double‐sided sticky tape next to the placement area. Any ticks that do not attach can be placed on the stick tape to prevent excape.

8 Exhale a couple of times into the inner Ziploc bag containing the vials with the nymphal ticks. This will stimulate the ticks, and from their movement you can gauge their readiness to feed.
  a. For *A. americanum* placement, apply a 2‐in. (∼5 cm) wide Elizabethan collar around the guinea pig's neck for nymphal tick placement on the head. *A. americanum* moves very fast, and the Elizabethan collar will prevent escape of the nymphs over the back of the guinea pig.
  b. The nymphs are easy to spot climbing up the Elizabethan collar and can be recollected with a fine paintbrush for replacement in the fur trenches.
    Note that A. americanum nymphs that have not settled within the first couple of minutes but have continuously moved around are not likely to bite.
9 Carefully unstop a vial containing nymphal ticks right over the shaven area on the anesthetized guinea pig. Keep the stopper close at hand to seal the vial again to stem excessive nymphal tick escape from the vial.
10 Using a fine paintbrush, collect nymphs from the holding vial and place them on the trimmed area on a sedated guinea pig. You may also use tweezers to gently grasp the tick about the idiosoma, placing the tick “face in” to the grain of the hair.
  a. In the case of *A. americanum*, place the nymphs into the cut fur trenches. To make that easier, first brush the fur against the grain so it stands up and opens the trenches.
  b. Importantly, unlike other nymphal tick species, *A. americanum* moves very fast. We recommend placing *A. americanum* nymphs more gradually (5 at a time) until you are more comfortable with the protocol.
  c. Try to spread out placed ticks over the trimmed area. This makes counting nymphal ticks easier, and it reduces multiple ticks impacting the same bite site.
  d. We also recommend placing more nymphal ticks than the desired final number of attached ticks. For example, if you aim for 15 attached nymphal ticks, place initially a total of 20 nymphal ticks.
  e. Note that nymphs ready to feed will move around a little and then start to settle on a spot. Uninterested nymphs will try to walk off the animal. For white mice (BALB/c and Swiss Webster), tick visibility is simple, but in black mice (C57BL/6), ticks are more difficult to see, and care must be taken to ensure that the ticks do not migrate to other body areas.
  f. In mice, gentle squeezing of the idiosoma with tweezers can stimulate the tick to expose its mouthparts, which can then be placed directly into the skin. Confirmation of attachment can be achieved by gently tugging on the tick or brushing against the grain of the hair. This manual embedding of the mouthparts is highly successful in mice but cannot be done in guinea pigs due to their tougher skin. When using this method, stubble is less necessary, and in this way, mice that have been depilated can still be infested.
11 Once placed, give the nymphs a moment to settle. With a fine paintbrush or tweezers, you may have to catch nymphal ticks that are moving off the target site and replace them on the shaven area.
12 Once the nymphs start to settle down, start dabbing them repeatedly, but gently with a fine paintbrush onto the rear of the nymphal ticks to encourage burrowing into the fur stubble. Continue dabbing the nymphs in regular intervals even after they burrow into the fur stubble.
  Continued dabbing accomplishes several things; for one, it prompts the nymphs to remain stationary. Secondly, they get closer to the guinea pig's skin, which promotes biting. Thirdly, burrowing into the fur stubble allows stimulation of dorsal sensilla on the nymphal tick, which promotes biting.
  Note that A. americanum does not like to be dabbed repeatedly. They respond better to brushing the longer fur between tranches side to side. The movement of the fur encourages most A. americanum ticks to burrow into the fur toward the skin. Note that A. americanum nymphs that have been exposed to sunlight long enough will readily burrow into the fur with little encouragement.
13 About 10 to 15 min after initial placement and dabbing, check for attachment by brushing against the grain of the fur stubble with a fine paintbrush.
  a. Unattached nymphs will pop out of the fur stubble when you brush against the grain. Some nymphs may sit in the fur stubble without attaching, and these should be removed and replaced with fresh nymphs.
  b. Do not brush against the grain with *D. variabilis*. *D. variabilis* is notoriously slow to attach, and most nymphs will be brushed out of the fur if you brush against the grain. Here, you must verify attachment ∼2 hr placement. We recommend placing *D. variabilis* in more generous excess.
  c. Brushing against the grain is less straightforward with *A. americanum*, as they sit in the fur trenches and they do not like to be dabbed a lot. Instead, brushing with a fine paint brush over the untrimmed hair against the grain will allow you to see movement of unattached nymphal ticks, while also stimulating unattached nymphs to bite.
14 Keep watching the nymphs for at least 30 min, while continuing to dab the nymphs intermittently.
  Do not dab A. americanum. Instead, brush the long hair occasionally against the grain to look down into the fur trenches and to continue to stimulate burrowing into the stubble.
15 Return the guinea pigs to their containment cages once they show signs of recovery from the injectable general anesthesia.
  a. Remove the Elizabethan collar placed on guinea pigs for *A. americanum* nymphal tick placement before their full recovery.
  b. Alternatively, place a 2‐in. (∼5 cm) wide Elizabethan collar around the neck of guinea pigs. This can be done either before recovery from the ketamine/xylazine anesthesia, or during the recovery from the isoflurane anesthesia during tick counting further below. Ensure that the Elizabethan collar is placed loosely enough to allow space for a small finger to prevent choking, but tight enough to prevent the guinea pig from removing the Elizabethan collar on its own. Guinea pigs exposed to *A. americanum* are already wearing Elizabethan collars.
  c. Note that the Elizabethan collar cannot have a rim >2‐in. (5 cm) wide, or the guinea pig will struggle to feed and move. We manually trim the Elizabethan collars down to the correct size with scissors.
16 Continue to monitor the guinea pigs until fully recovered from anesthesia.
  If Elizabethan collars are placed, note that guinea pigs tend to have a violent reaction to the Elizabethan collar during the first 10 to 15 min after recovery. They will try to remove the Elizabethan collars by thrashing and scratching. Do not be alarmed.

#### Confirm nymphal tick attachment and adjust attached nymphal tick numbers

Nymphal ticks may require 2 to 3 hr after placement to be embedded firmly enough to preclude spontaneous detachment and moving off placement location or even dropping off. Rechecking nymphal tick attachment 2 to 3 hr after placement also serves to adjust attached nymphal tick numbers on all guinea pigs to the same number of attached ticks. This part of the protocol can be managed by a single person.

As multiple ticks are attached per guinea pig, guinea pigs become a significant source of data variability further enhanced by varying behavior between individuals. Having the same number of attached ticks at the beginning of a tick removal experiment helps balance the weight of data points during the analysis.

17 Anesthetize tick‐exposed guinea pigs 2 to 3 hr after initial nymphal tick placement with isoflurane. Induction of anesthesia takes 2 to 3 min, depending on the weight of the guinea pig.
  a. Use 4% isoflurane at 2 L/min flow rate with a conventional isoflurane vaporizer and at 1 L/min flow rate with a SomnoFlo.
  b. Alternatively, if Elizabethan collars were placed after tick placement, depending on the size of your induction chamber, remove the Elizabethan collar before placing the guinea pig in the induction chamber. Otherwise, remove the Elizabethan collar after induction of anesthesia, which is often the easier option.
18 Place the guinea pig on a blue pad and place an appropriately sized nose cone over the guinea pig's snout to maintain isoflurane anesthesia.
19 Confirm tick attachment by brushing against the grain with a fine paint brush.
  a. With *A. americanum*, you can trim the longer fur now that was left to form the fur trenches with electric contour clippers. Hold the clipper blade flat against the guinea pig, which raises the cutting edge 1 to 2 mm above the skin, which will prevent cutting the nymphal ticks.
  b. Move the clippers against the grain from the neck area toward the nose. Trimming the long fur will make it easier to count attached ticks, as it will also reveal nymphs attached within the longer fur.
  c. Once the fur is trimmed, confirm nymphal tick attachment by brushing the stubble against the grain.
20 Count the attached ticks and remove excess attached nymphal ticks.
  a. Use curved fine‐tipped tweezers to remove the attached nymphal tick. Grab them with the tweezers as close to the skin as possible and then remove the nymphal ticks by gradually pulling them out perpendicular to the skin (do not twist the tweezers or pull at an angle as that may tear off the hypostome).
  b. We recommend conducting tick exposures with 10 to 15 attached nymphal ticks to build a big enough *N* for data analysis. Try to adjust the number of attached nymphal ticks to the same number for all guinea pigs to balance the weight each tick will carry as a data point for data analysis.
  c. Note that guinea pig sensitization can be accomplished with a single bite (Doehl et al., 2026). But it is more effective to have at least 5 nymphal ticks attached to account for spontaneous loss due to random tick removal during routine grooming.
  d. Alternatively, place a 2‐in. (∼5 cm) wide Elizabethan collar around the neck of guinea pigs. Ensure that the Elizabethan collar is placed loosely enough to allow space for a small finger to prevent choking, but tight enough to prevent the guinea pig from removing the Elizabethan collar on its own.
21 Return the guinea pigs to their containment cages.
22 Continue to monitor the guinea pigs until fully recovered from anesthesia.
  If Elizabethan collars are placed, note that guinea pigs tend to have a violent reaction to the Elizabethan collar during the first 10 to 15 min after recovery. They will try to remove the Elizabethan collar by thrashing and scratching. Do not be alarmed.

## Basic Protocol 2 ASSESSING EFFICACY OF ITCH‐INDUCED TICK REMOVAL BY QUANTIFYING LOSS OF TICKS OVER TIME

One of the key observations in the study of itch‐induced tick removal is the increasing speed and frequency with which sensitized guinea pigs remove attached ticks from their bodies. This protocol follows on from a successful tick placement described in Basic Protocol 1.

We recommend that all animals should have the same number of ticks, as this will give each tick the same statistical weight. To quantify tick loss, select specific time points post tick placement on which to assess tick numbers. For data collection, count ticks that remain on each guinea pig at these specific time points post tick placement.

### Materials

Guinea pigs (from Basic Protocol 1)
Isoflurane (e.g., “Forane” from Baxter)

Elizabethan collars (Rat from Kent Scientific, cat. no. EC404VL‐10)
Isoflurane vaporizer (e.g., from Vetequip or “SomnoFlo” from Kent Scientific)
Fine‐tipped paintbrush
Fine‐tipped, curved tweezers (e.g., Dumont #7 forceps from FST, cat. no. 11272‐50)
Notepad and pen

#### Placement of ticks from guinea pigs

1 If using nymphal ticks, follow Basic Protocol 1 for tick exposure.
  a. Each guinea pig must be placed in individual triple‐containment cages to prevent tick removal by social grooming.
  b. If you wish to disrupt tick removal, then place an Elizabethan collar around guinea pigs’ necks with ticks on their heads.

#### Counting of attached ticks

2 At desired time points post‐tick placement, anesthetize guinea pigs with isoflurane for 2 to 3 min in an induction chamber, and place the guinea pig afterward on a noise cone for continued anesthesia.
  a. Use 4% isoflurane at 2 L/min flow rate with a conventional isoflurane vaporizer and at 1 L/min flow rate with a SomnoFlo.
  b. Note that we strongly recommend the use of isoflurane when you are new to the protocol. This way you can assess general attachment with a small paintbrush, look at individual ticks more closely with a magnifying glass, and count with more control. For larvae counting, anesthesia is a must. For nymphs and adults, once you are more familiar with the protocol you may trust your judgment.
3 Count the number of attached ticks per guinea pig.
  Count attached ticks by looking at the guinea pig from behind. This way it is easier to look between the fur stubble and see the attached ticks. Record the data using a notepad and pen.
4 Return guinea pigs to their respective triple‐containment cage.
  After the tick counts are taken at the final time point, remove all the remaining ticks from the guinea pigs under isoflurane anesthesia. Tick removal is a painful process, particularly adult ticks and Amblyomma nymphs, and the anesthesia will reduce discomfort for guinea pigs and mice alike.
5 Repeat steps 2 to 4 for each time point you wish to count attached ticks.

#### Converting tick counts to time‐to‐event (survival) data

6 Generate time‐to‐event data from count data.
  Instead of using the counts of remaining ticks per guinea pig for the data analysis, it is more effective to convert the data into time‐to‐event data, also known as survival data, as we want to estimate the probability of a tick being attached or removed at a given time point. In the data table structure for time‐to‐event data, each tick in the study represents a row. For example, if you use 6 guinea pigs in an experiment and each guinea pig bears 15 ticks at the start of the experiment, then the data table will have 90 rows (6 × 15 = 90). Make sure to group ticks by guinea pig, as you will need the information on how many ticks were removed by each guinea pig for the data analysis. Thus, the first 15 rows of your data table are dedicated to the ticks from guinea pig 1. The ticks from row 16 to 30 belong to guinea pig 2, etc. When a tick is found to be removed at the time of tick counting, a “1” is placed for that tick in the data column. Note that it does not matter which tick in the 15‐row block for a guinea pig receives the 1. However, ensure that the corresponding time stamp of observed removal is recorded in the same row as the *x*‐value. Should a tick still be attached at the end of data collection, then a “0” is placed in the data column with the time of the final time point in the time column. The secondary effect of converting your count data into time‐to‐event data is that you dramatically increase your *N*. Instead of having 6 tick counts for the six guinea pigs used, you have 90 data points for the total number of ticks in the study, which increases statistical power. On the flipside, guinea pigs become a source of data variability, which increases the complexity of the data analysis.
7 Once the dataset is complete, refer to the statistics section of this protocol for analysis.
  We recommend the use of the Cox Proportional Hazards Model.

## Basic Protocol 3 ASSESSMENT OF HEALTH/ABILITY OF PARTIALLY FED NYMPHAL TICKS TO FEED DETERMINED BY SCUTAL INDEX

In the literature, an adult female tick's ability to feed and its overall health are often assessed by measuring its engorgement weight, the weight of the produced egg mass, and the eggs’ hatching success (Gebbia et al., 1995; Owen et al., 2025). However, if your experimental design does not allow for feeding completion, and/or you are using larvae or nymphs, these metrics are inapplicable. As an alternative measure, we adapted the use of the scutal index (Meiners et al., 2006). During tick feeding, the alloscutum (i.e., the region posterior to the scutum) expands over time, while the scutum remains unchanged. The quotient of alloscutum length and scutum width increases as the tick feeds, allowing a robust measure of a tick's ability to feed at any given time point during the feeding process irrespective of the analyzed tick developmental stage.

### Materials

Guinea pigs (from Basic Protocol 1)
Isoflurane (e.g., “Forane” from Baxter)
Petroleum jelly (e.g., Vaseline)

Elizabethan collars (Rat from Kent Scientific, cat. no. EC404VL‐10)
Isoflurane vaporizer (e.g., from Vetequip or “SomnoFlo” from Kent Scientific)
Medical blue pads
Fine‐tipped, curved tweezers (e.g., Dumont #7 forceps from FST, cat. no. 11272‐50)
Small specimen vials
Clear or white double‐sided sticky tape
Small white tray (at least 4 × 4–in. [10 × 10–cm])
Stereomicroscope with camera (e.g., Leica M165 FC stereomicroscope with a Leica DFC 7000 T camera)
Dual gooseneck microscope illuminator
Fine‐tipped paintbrush

#### Placement and collection of ticks from guinea pigs

1 If using nymphal ticks, follow Basic Protocol 1 for tick exposure and place an Elizabethan collar around guinea pigs’ necks with ticks on their heads.
2 For tick collection, anesthetize guinea pigs with isoflurane for 2 to 3 min in an induction chamber at desired time‐points post tick placement.
  Use 4% isoflurane at 2 L/min flow rate with a conventional isoflurane vaporizer and at 1 L/min flow rate with a SomnoFlo.
3 Remove the Elizabethan collar and place the guinea pig on a blue pad. Place a nose cone over the guinea pig's snout to maintain isoflurane anesthesia.
4 Remove attached ticks by grabbing them with curved tweezers as close to the skin as possible and then remove the tick by pulling it perpendicular to the skin.
  Do not twist the tweezers or pull at an angle, as that may tear off the hypostome.
5 Collect removed ticks in a 5‐ml specimen tube or similar.
  a. You can either collect all attached ticks, or you can collect only a subset of ticks, leaving the remainder to continue feeding for later collection. If the latter approach is used, replace the Elizabethan collar after tick collection.
  b. The latter is statistically more robust as the data is paired between time points, but it will increase the complexity of the statistical analysis.
  c. Guinea pigs are a potential source of data variability. Therefore, we recommend that ticks from each guinea pig are collected in separate tubes.
6 Return the guinea pigs to their containment cages (if ticks remain attached) or their home cage for recovery from the anesthesia.
7 Continue to monitor the guinea pigs until fully recovered from anesthesia.

#### Imaging of collected ticks

8 Glue a piece of double‐sided sticky tape on the bottom of a small white tray.
  Try to place the tape as flat as possible and avoid air bubbles.
9 Apply a thin layer of petroleum jelly on the rim of the tray.
10 Open the specimen tube over the tray and remove individual ticks with fine‐tipped tweezers.
  If needed, CO_2_ can be used to stun the ticks, which makes collecting them one‐by‐one from the tube easier. Alternatively, the collection tube can be chilled on ice to produce a cold shock, but prolonged cold exposure may kill the ticks (avoid placing directly on ice; separate the ice from the ticks with cloth or paper towel).
11 Place the ticks onto sticky tape. Space them apart for easier imaging.
  Ensure that the ventral side of the tick is glued down and that the dorsal side is fully visible from the top. Otherwise, it will be impossible to measure the scutum's width accurately.
12 Once all ticks from a specimen vial are stuck on the tape, move the tray underneath a stereomicroscope.
  We used a stereomicroscope from Leica (M165 FC) with a Leica DFC 7000 T camera (Leica, IL) using the Leica Application Suite X v.3.7.4.23463. However, any equivalent setup will do.
13 Adjust a dual gooseneck microscope illuminator for optimal illumination of the ticks on the tray.
14 Zoom into the first tick until the tick fills the field of view. Adjust the lens of the stereomicroscope until the scutum is in focus.
  At this point, the user may assess the ticks for signs of life. Live ticks will typically show signs of leg movement, in particular, from the front legs. If needed, ticks can be stimulated by dabbing them gently with a fine paintbrush.
15 Take an image.
  It is recommended to maintain the same degree of magnification between ticks for consistency in case the images will be used for visual assessments.
16 After imaging all ticks, either dispose of the ticks safely, or re‐collect them for further processing.
  If ticks are not processed for other experiments post imaging, then secure the ticks on the tape by gluing another piece of tape on top, so that the ticks are sandwiched between the tapes. Remove the tape with the ticks from the tray and secure it in a Ziploc bag. Place the bag in a freezer for at least 24 hr before disposing of it in accordance with your institution's guidelines. In our case, we move the bag into a medical waste container for incineration.

#### Measure the scutum width and alloscutum length

**Figure 3:**
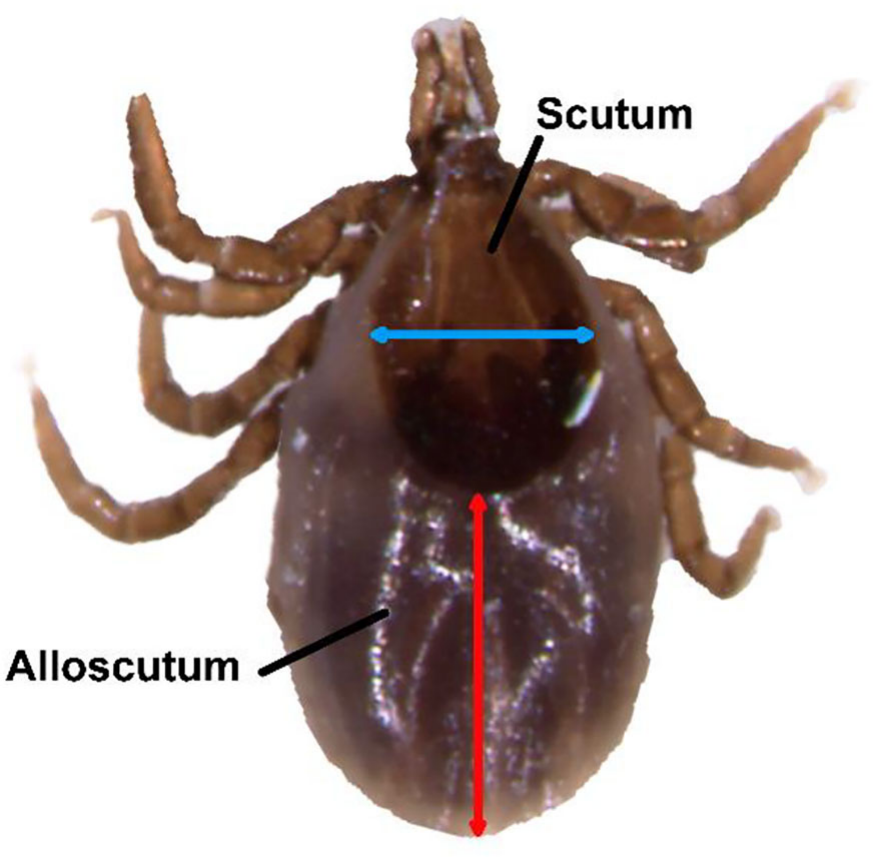
Graphical representation of where to measure alloscutum length and scutum width to calculate the scutal index.

17 Load the images into a software package with a measuring tool for length.
  We use the Leica Application Suite X that is also used for image acquisition. However, other tools can be used, like ImageJ. Make sure to export images from the acquisition software in a supported uncompressed format for programs like ImageJ. If you need your measurements in, for example, µm, acquire an image with the same stereomicroscope setup and magnification of a ruler or something that shows a known distance to calibrate ImageJ. Otherwise, the software may not be able to give you accurate measurements in a scale of distance (e.g., µm), but only pixel counts. However, if you only want the scutal index, you can also work with the default pixel counts to produce your ratios, as these will be the same whether you use pixel counts or µm post‐conversion.
18 Measure alloscutum length from the edge of the scutum to its rear end and scutum width at its broadest point (Fig. 3).

19 Calculate the scutal index as follows:
$$Scutal\;index=\frac{Alloscutum\;length}{Scutum\;width}$$
  This way, the scutal index will increase as the tick engorges. Note that the scutal index has no scale and is simply a ratio to be compared relatively to another group.

## Basic Protocol 4 SCRATCH BOUT QUANTIFICATION IN GUINEA PIGS

**Figure 4:**
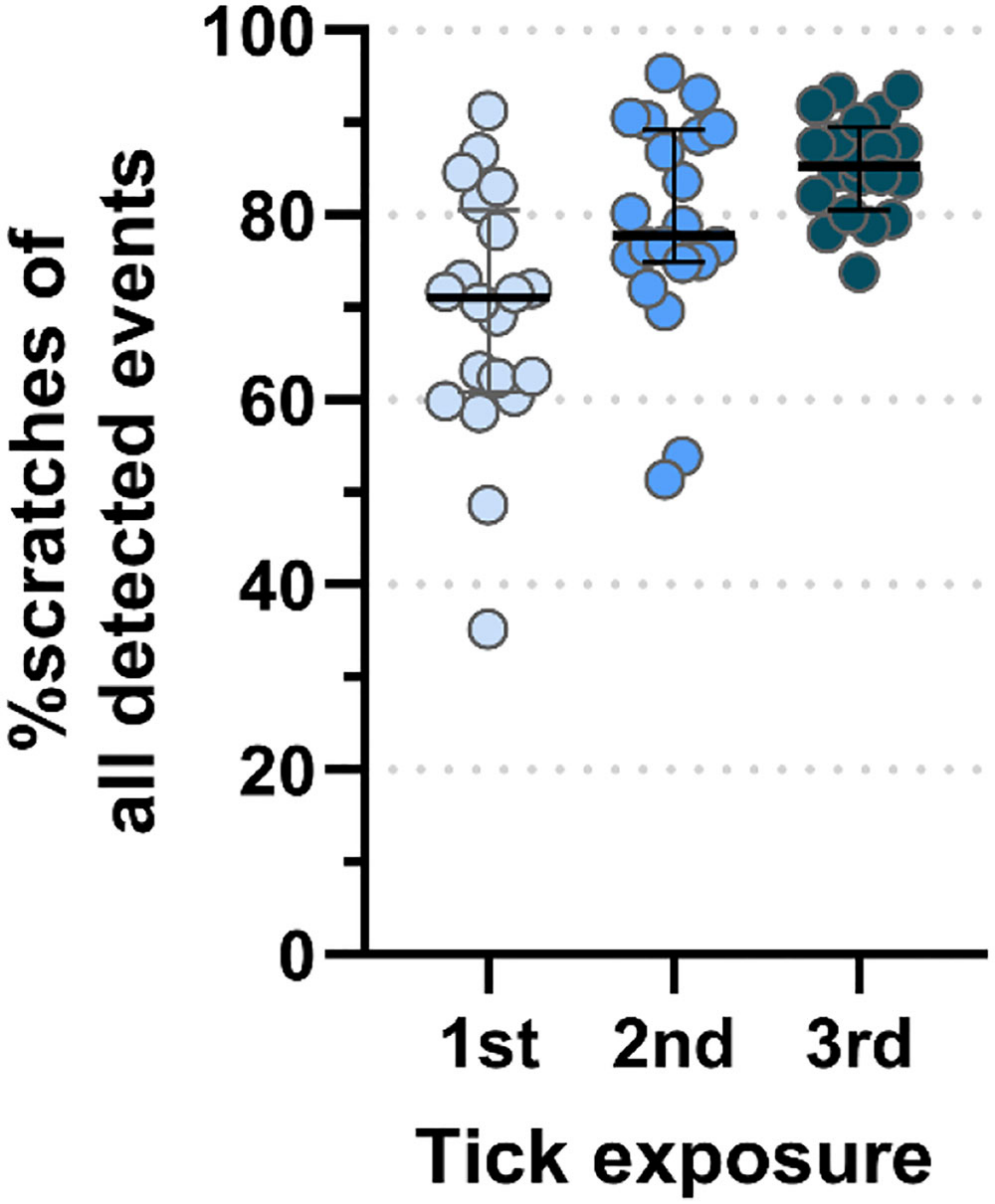
Scatter plot of percent correctly identified scratch bouts for the 1^st^, 2^nd^, and 3^rd^ tick exposure in guinea pigs.

A crucial variable to measure for the study of itch‐induced tick removal is scratching. Scratching is a proxy measure for itch and itch intensity in the scientific literature (Erickson & Kim, 2019; Felix & Shuster, 1975). Our data showed a clear correlation between the intensity of scratch activity at the tick bite site and the number of ticks that remain attached per time point (Doehl et al., 2026). As guinea pigs tend to freeze in the presence of a perceived threat, which includes humans, scratch bouts can be observed directly, but not reliably. Instead, guinea pigs should be recorded in the absence of humans. Overnight recording with infrared cameras is highly recommended. With guinea pigs, nighttime will not affect their behavior compared to daytime. Guinea pigs are generally considered crepuscular (i.e., most active during twilight hours). However, guinea pigs sleep in short bouts lasting seconds to up to 10 min around the clock, giving them a relatively even activity profile around the clock. As recordings can be lengthy, we developed an automated scratch bout counting app that can process the recordings (Support Protocol). The use of the app greatly accelerates the analysis of recordings. While the app is quite accurate (Fig. 4), we recommend visual confirmation of scratch bout classification post‐app analysis.

### Materials

Guinea pigs (from Basic Protocol 1 or not tick exposed)
Petroleum jelly (e.g., Vaseline)

Recording cameras and cages (e.g., the PhenoTyper system from Noldus)
Computer workstation with multi‐core CPUs (16+ cores recommended), 64 GB system memory, and a dedicated GPU (an A100‐level GPU recommended)
Access to a supercomputing cluster for long wall clock time and parallel processing of multiple recordings (preferably)
Python version 3.12 or greater
The Tick‐Scratch Detection Tool, available at: https://github.com/IDSS‐NIAID/tick‐scratch‐detection

#### Placement of nymphal ticks from guinea pigs

1 Follow Basic Protocol 1 for nymphal tick exposure.
  You can also assess scratch bouts on unexposed guinea pigs for baseline acquisition.

#### Recording of guinea pigs

For recording guinea pigs, we employed the PhenoTyper system from Noldus and their proprietary recording software MediaRecorder. However, any camera setup that can film a guinea pig from above in an isolated cage and produces a file in the MP4 format can technically be used. It is very important to optimize the contrast between guinea pigs and cage background for optimal data collection. We use custom‐made absorbent cage liners (top layer: 85 grams per square meter (gsm) brushed/knitted polyester; middle layer: 250 gsm polyester soaker; bottom layer: 85 gsm brushed/knitted polyester bottom with waterproof TPU finished top) dyed with an infrared absorbent dye to appear black under the infrared camera.

2 Start the recording.
3 Place your guinea pigs into a Phenotyper cage setup.
  a. If the guinea pigs are tick‐exposed, we recommend using petroleum jelly around openings of the cage to prevent tick escape.
  b. Depending on the length of recording, it may be necessary to supply the guinea pigs with food, water, and cage liners for bedding that absorb urine.
4 Leave the room as quickly and quietly as possible.
  Important: Guinea pigs respond to danger by becoming completely immobile. The presence of humans in the same room during recording may interfere with the guinea pigs’ behavior and bias your data.
5 End the recording after a desired length.
  We record between 12 and 14 hr. Scratch bouts may be few and far between. Therefore, recording longer improves data acquisition. However, it also increases file size and will increase the demand for computing power for analysis.
6 Remove the guinea pigs from the Phenotyper cages and return them to the triple‐containment cages if they still have ticks attached. If not, return them to their home cages.

#### Recording analysis

We developed an automatic pipeline based on a trained deep learning neural network to detect scratch events in the recordings. The app consists of a set of Python functions that need to be called manually to process the recordings. Details on the inner workings of the app can be found in Support Protocol below. The app is currently set up to function in the Bash environment of a computing cluster. For the proper execution of the app, a Python environment must be created within the Bash environment. However, with appropriate modifications, the app can be hosted with a Python environment on a personal computer. Recording analysis requires a lot of computing power: the longer the recordings are, the more computing power is needed. Thus, only short recordings are recommended for processing on a personal computer.

7 Upload the app files onto your computing cluster.
8 Upload the MP4 files to the computing cluster.
  We recommend loading the videos into a subdirectory of the main directory where also the app scripts are stored.
9 Set up the Python environment in which to run the app on your computing cluster.
10 The execution of the app commences in 5 steps:
  a. Step 1: Amend the input and output directories in the slurm submission file 1_fix_duration in the line “python fixDuration.py *input_folder_directory output_folder_directory*”, then execute the file.
    i. The input folder is the directory into which the raw MP4 files were loaded to the computing cluster.
    ii. The output folder is the new directory into which the modified files are copied. If input and output folders are identical, the raw files will be overwritten by the modified files. We recommend avoiding that in case there is a need to return the raw MP4 files.
    iii. Script 1_fix_duration fixes duration parameters of our recording files. If unresolved, this issue will cause frame parsing in OpenCV and stop the app prematurely. Since this is an artifact of MediaRecorder, it may not apply to your files.
    iv. In a Bash environment, set up the Python environment, then execute the script 1_fix_duration by calling “sbatch *directory of the app*/1_fix_duration”. The script will automatically iterate through all recordings in the input folder.
    v. Note that it is recommended to avoid white spaces in your directory names. If there are white spaces, put the whole directory into quotation marks (“*directory address*”), or use the backslash (\) to *escape* the white space. For example, “. ./data/raw data/mp4 videos/” becomes “. ./data/raw\ data/mp4\ videos/”.
  b. Step 2: Amend the input and output directories in the slurm submission file 2_motion_frames script in the line “python getMotionFramesMultiThreadCV2.py *input_folder_directory output_folder_directory*”, then execute the script.
    i. The input folder directory for step 2 is equivalent to the output directory from Step 1.
    ii. The output folder is the new directory into which the modified files are copied. It is recommended to create a new directory for the output to avoid any issues with file calling in the subsequent steps.
    iii. Script 2_motion_frames identifies and pulls out all the frames that show sufficient pixel changes from dark to bright and vice versa from the previous frame to identify motion of the guinea pig.
    iv. In a Bash environment, set up the Python environment, then execute the script 2_motion_frames by calling “sbatch *directory of the app*/2_motion_frames”. The script will automatically iterate through all recordings in the input folder.
    v. Note that it is recommended to avoid white spaces in your directory names. If there are white spaces, put the whole directory into quotation marks (“*directory address*”), or use the backslash (\) to *escape* the white space. For example, “. ./data/raw data/mp4 videos/” becomes “. ./data/raw\ data/mp4\ videos/”.
  c. Step 3: Execute the script 3_framePredict_array_job.py.
    i. The input folder directory for Step 3 is equivalent to the output directory from Step 2.
    ii. No new output folder is specified here.
    iii. Script 3_framePredict_array_job.py classifies the extracted frame series from Step 2 as either “scratch” or “non‐scratch”.
    iv. Note that this step is written as a Slurm array job and requests one A100 GPU per recording analyzed. Thus, the app currently assumes it operates on a high‐performance computer cluster. This will require re‐coding to work on a personal computer.
    v. In a Bash environment, set up the Python environment, then execute the script 3_framePredict_array_job.py by calling “python 3_framePredict_array_job.py‐root ″*output directory of step 2*”. The script will automatically iterate through all recordings in the input folder.
    vi. Note that it is recommended to avoid white spaces in your directory names. If there are white spaces, put the whole directory into quotation marks (“*directory address*”), or use the backslash (\) to *escape* the white space. For example, “. ./data/raw data/mp4 videos/” becomes “. ./data/raw\ data/mp4\ videos/”.
  d. Step 4: Amend the input and output directories in the slurm submission file 4_positive_clips in the line that commences with “python detectPositiveClips.py”, then execute the script.
    i. The input folder following “‐predict_root” is the output folder from Step 3 (equivalent to the output folder from Step 2).
    ii. The input folder following “–video_root” is the output folder from Step 1.
    iii. The output folder following “–clip_output” is a new directory into which the recording clips of identified scratch events are copied.
    iv. 4_positive_clips identifies frame series that were classified as scratch and compiles them into short recording clips.
    v. In a Bash environment, set up the Python environment, then execute the 4_positive_clips script by calling “sbatch *directory of the app*/4_positive_clips”. The script will automatically iterate through all recordings in the input folder.
    vi. Note that it is recommended to avoid white spaces in your directory names. If there are white spaces, put the whole directory into quotation marks (“*directory address*”), or use the backslash (\) to *escape* the white space. For example, “. ./data/raw data/mp4 videos/” becomes “. ./data/raw\ data/mp4\ videos/”.
  e. Step 5: Manually assess all recording clips to verify that they are correctly classified as scratch bouts.
    Although quite accurate (Fig. 4), the recording analysis app's identification of scratch bouts is not without fail. Therefore, visual confirmation of correct classification of scratch bouts is essential.

## DEVELOPMENT OF A SEMI‐SUPERVISED DEEP LEARNING PIPELINE FOR AUTOMATED DETECTION AND VALIDATION OF SCRATCHING BEHAVIOR

Most commercially available recording analysis software packages, including EthoVison XT from Noldus, which we procured together with their PhenoTyper System, are optimized for rodents with tails (e.g., mice and rats). Guinea pigs have no visible tail, which proved to be a significant problem for behavioral analysis in the software we tested. The software was unable to accurately detect the rear of the guinea pigs. Manual analysis was not feasible due to the length of our recordings (12 to 14 hr). Therefore, we developed an automated recording analysis app based on a deep learning neural network. This Support Protocol describes the development of our app. By necessity, the protocol is kept technical for brevity and is directed at users with a robust foundation in Python coding, like bioinformaticians. Users may follow these steps to recreate our app, further optimize it, or adapt it to a different platform. Our recording analysis app is currently optimized to run in the bash environment of a computing cluster. It is recommended to analyze long recordings with a computing cluster for increased computing power to accelerate the process. The recording analysis app files are available for download at: https://github.com/IDSS‐NIAID/tick‐scratch‐detection.

### Materials

Python version 3.12 or greater
Computer workstation with multi‐core CPUs (16+ cores recommended), 64 GB system memory, and a dedicated GPU (an A100‐level GPU recommended)
Access to a supercomputing cluster (preferably)

1 Metadata standardization.

To ensure compatibility with computer vision libraries and prevent data truncation, raw recording files (MP4 format) underwent a metadata correction process. FFmpeg lossless copying (‐codec copy) to a new file container was applied to each raw recording while explicitly ignoring the edit list atom (‐ignore_editlist 1). This step corrected discrepancies in the duration property, ensuring that OpenCV could read all frames without premature termination.

2 Motion detection and frame extraction.

Frames containing motion were detected using a frame‐differencing pipeline implemented in Python (v3.12) using the OpenCV saturated subtraction (cv2.subtract(prev, current)). This unidirectional operation isolates regions transitioning from light to dark. In the context of our dataset, which features bright objects moving against a dark background, this captured the trailing edges of the moving targets, generating consistently sufficient pixel activation to trigger the motion threshold. For each recording, the frame sequence was processed iteratively. A difference map was generated by subtracting the intensity values of the current frame from the previous frame. A median blur filter (kernel size 3) was applied to the difference map to remove noise. Subsequently, an adaptive thresholding operation (Adaptive Gaussian Thresholding) was performed with a block size of 11 and a constant subtraction value of 3 to generate a binary motion mask.

Connected component analysis was applied to the binary mask to filter out insignificant changes. Components with a calculated area of ≤81 pixels (9 × 9) were classified as noise and removed. If the remaining mask contained any non‐zero pixels, the corresponding raw frame was designated a “motion frame” and saved as a JPEG image. The filename of each saved image encoded the absolute frame index to preserve temporal information.

3 Unsupervised feature learning and training data generation.

To generate a labeled dataset for the training of the behavioral classifier without requiring manual annotation of 14‐hr‐long recordings, a semi‐supervised clustering approach was employed.

Region of Interest (ROI) Extraction: To facilitate efficient processing of the long‐duration input recordings (14 hr), a high‐throughput computer vision pipeline was selected over heavy foundation models to localize the animal. While the Segment Anything Model (SAM) was evaluated for automated subject segmentation, it was rejected due to excessive computational latency when scaled to full‐length recordings. Consequently, a heuristic approach based on luminance and geometric properties was implemented to isolate the largest bright object in the scene.

On each frame, Otsu's thresholding method was used to separate foreground objects. Because of the grayscale nature of the frames, Otsu's thresholding is applied to the first (red) channel only. To prevent under‐segmentation in low‐contrast conditions, a minimum threshold floor of 120 intensity units was enforced. A binary mask was generated, and the properties of connected regions were analyzed. The region exhibiting the largest area was identified as the subject. A bounding box was constructed around this region and padded by 30 pixels in all directions. To satisfy convolutional network input requirements, the bounding box was squared (expanded to match the largest dimension), and the image content was cropped and resized to 256 × 256 pixels using inter‐area interpolation. If the padded coordinates exceeded image boundaries, the crop window was shifted to remain within the frame.

Convolutional Autoencoder (CAE) Training: A CAE model was trained to learn a 128‐unit latent representation of the guinea pig subjects. The network architecture uses a standard symmetric encoder‐decoder structure. The encoder has four convolutional blocks; each block contained a 2D convolutional layer (3 × 3 kernel, ReLU activation, “same” padding) followed by a max‐pooling layer (2 × 2). The filter depths for the four blocks were 32, 16, 8, and 4, respectively. The resulting feature map was flattened and passed through a dense bottleneck structure including fully connected layers with 512, 128, 512, and 1024 units, before being reshaped to a 16 × 16 × 4 tensor. The decoder mirrored the encoder using four blocks; each uses a transposed convolutional layer (stride 2) for upsampling, followed by a standard convolutional layer to refine features. The decoder output layer uses a single filter with a sigmoid activation function to reconstruct the grayscale input. The input data, consisting of extracted ROIs, was split into training (85%) and testing (15%) subsets. To improve model robustness, data augmentation was applied during training, including random brightness and contrast adjustments (factor range 0.75 to 1.25), horizontal and vertical flipping, and rotation (0 to 360 degrees) with translation (±25 pixels). The model was trained for 100 epochs using the Adam optimizer with a learning rate of 1 × 10^−4^ and Mean Squared Error (MSE) reconstruction loss. Training was monitored using callbacks for model checkpointing, early stopping (patience of 7 epochs), and learning rate reduction (factor 0.1, patience of 3 epochs).

Feature Space Clustering: Following the CAE training, the encoder part was utilized to extract latent feature vectors from the raw frames. These high‐dimensional features were projected into a 32‐dimensional space using Principal Component Analysis (PCA). Subsequently, Uniform Manifold Approximation and Projection (UMAP) was applied to the PCA‐reduced features to generate a low‐dimensional embedding suitable for density‐based clustering. HDBSCAN (Hierarchical Density‐Based Spatial Clustering of Applications with Noise) was performed on the UMAP embeddings with a minimum cluster size of 100 samples. This unsupervised process grouped visually similar animal postures. The resulting clusters were manually inspected, and frames were binary labeled as either “positive” (scratching) or “negative” (non‐scratching) to form the ground truth training set.

4 Supervised classifier training.

A binary classification model was trained using the labeled dataset generated via the clustering pipeline. The dataset was split into training (80%), validation, and a fixed test set (1024 samples per class). To address class imbalance between scratching and non‐scratching frames, the training and validation sets were oversampled to match the count of the majority class.

The network architecture utilized an EfficientNetB4 backbone pre‐trained on ImageNet weights. The top classification layers of the base model were removed. Features were extracted from the third‐to‐last layer of the base model and passed through a Global Average Pooling 2D layer, followed by a fully connected dense layer with a single output unit utilizing a sigmoid activation function. Transfer learning was implemented by freezing the initial layers of the backbone and only fine‐tuning the final 50 layers.

The model was trained using the RMSprop optimizer (learning rate 1 × 10^−5^) and binary cross‐entropy loss for 150 epochs. Data augmentation techniques, including random brightness, contrast, flipping, and rotation, were applied during training. Model performance was monitored via validation loss, with early stopping (patience 7) and learning rate reduction (patience 3) employed to prevent overfitting. The final model was evaluated using the Area Under the Receiver Operating Characteristic Curve (AUC–ROC) on the independent test set (AUC score = 0.99).

5 Inference: Region of interest extraction and behavioral classification.

The trained deep learning pipeline was applied to the full dataset to classify the extracted motion frames and detect the occurrence of scratching behavior. The ROI extraction operation described in step 3 was applied to input recording frames to generate inference input data.

Inference: The EfficientNetB4 model weights (trained as described in step 4) were loaded. ROI images were processed in batches of 64. The network output a probability score (0.0 to 1.0) for each frame, representing the likelihood that the frame contained a scratching event. These scores were serialized to pickle files (.pkl) associated with their source recordings.

6 Temporal clustering and event clip generation.

The final generation of positive event clips was performed by analyzing the temporal distribution of the prediction scores. A filtering and clustering algorithm was applied with the following logic:

(I) Thresholding: Frames with a prediction score exceeding 0.75 were flagged as positive (scratching) frames.
(II) Temporal clustering: Positive frames were grouped into continuous events. Event boundaries were defined when the gap between consecutive positive frame indices exceeded 50 frames.
(III) Duration filtering: Event clusters spanning fewer than 15 frame indices were discarded to eliminate transient false positives.

For every valid cluster, the start and end timestamps were calculated based on the frame rate (FPS), with a buffer of 1 s added to both the start and end times (–1 s and +1 s, respectively). The final recording clips were generated using FFmpeg. The relevant temporal segments were extracted and saved using the copy codec (‐c copy) to prevent re‐encoding degradation.

7 Interactive event validation and annotation interface.

To facilitate manual verification and refinement of the automated event detections, a custom web‐based application was developed using the Streamlit framework. The application was designed to interface directly with cloud‐based infrastructure, retrieving the generated MP4 clips dynamically from an Amazon Web Services (AWS) Simple Storage Service (S3) bucket via the s3fs and boto3 libraries.

The user interface was structured hierarchically, allowing operators to filter data by “Project” and “Trial” identifiers. Upon selection of a specific trial, the system queried a non‐relational database (AWS DynamoDB) to retrieve existing annotation metadata; if no prior records existed, a default label set was instantiated. The interface rendered the event clips in a responsive grid layout, utilizing “HTML5 video players” with autoplay and loop functionality enabled to permit rapid visual assessment. Each recording element was paired with a radio button input widget, allowing the user to assign discrete behavioral labels (as defined in the external configuration).

To optimize annotation throughput, the application allows the global assignment of a specific class label to all events within the current view or trial. All annotation updates were committed transactionally to the DynamoDB table, preserving the labeling state and a lastModifiedDateTime timestamp for version control. Finally, the system included a data serialization module utilizing the Pandas and OpenPyXL libraries to aggregate validated events. This module generated downloadable Microsoft Excel (.xlsx) reports containing precise temporal metadata (including trial identifiers, start timestamps, end timestamps, calculated event durations, and confirmed classification labels) for downstream statistical analysis.

#### Discussion

While the current pipeline relies on frame‐level spatial features to identify scratching behaviors, we acknowledge that scratching is inherently a temporal event defined by specific motion dynamics. The decision to utilize a 2D CNN architecture was driven by the computational efficiency required for high‐throughput screening and the initial scarcity of labeled recording sequences. A primary limitation of this approach is the potential for false positives from other postures like scratching, e.g., grooming. However, the semi‐supervised pipeline implemented here serves as a critical foundation for advanced temporal modeling. The dataset of validated events generated by this study will facilitate the future training of more advanced models, such as recurrent neural networks or transformer‐based recording classifiers, enabling the distinction between fine‐grained behaviors, such as tick‐scratching vs non‐specific grooming with high temporal resolution.

## Basic Protocol 5 ASSESSMENT OF LOCAL SKIN INFLAMMATION DUE TO IMMUNITY TO TICK BITES USING LASER SPECKLE CONTRAST IMAGING

Non‐invasive techniques are highly beneficial when working with animal models. They significantly reduce the number of animals required to conduct a study, prevent wastage of life, eliminate unnecessary discomfort to animals, and reduce costs. They permit repeated measures in the same animals over time and the application of different experimental techniques on the same animals, increasing the robustness and integrity of research data. Laser speckle contrast imaging is a non‐invasive tool that detects the amount of light refraction per pixel area. Essentially, the more cells move, the more variability in the light scattering pattern is detected. This way, blood flow can be visualized against the relative steadiness of cells in a tissue, like the skin. Interestingly, laser speckle contrast imaging can also detect the active immune cell influx into the skin. Immune cells that egress from the blood vessels into the dermal tissue are not stationary but are actively moving around, although in a more confined space compared to the lumen of a blood vessel. This motion is sufficient to generate signal contrast against the comparatively stationary skin cells (keratinocytes, fibroblasts, stromal cells, etc.). We found a good correlation between the observed amount of cell influx into the tick bite site by histology and laser speckle imaging for most tick species within limitations. Once the density of immune cells in the tick bite site reaches a high level, the motion of these cells is reduced as they pack together tightly. This is observed with, for example, *R. sanguineus* at 48 hr post tick attachment (pta). Even so, clear differences between treatment and control groups have been observed when using laser speckle contrast imaging.

### Materials

Guinea pigs (from Basic Protocol 1)
Isoflurane (e.g., “Forane” from Baxter)
Depilatory cream (e.g., Nair or Veet)
Water
Anesthesia (see recipe)
Lubricant eye ointment (e.g., “Systane” from Alcon)

Isoflurane vaporizer (e.g., from Vetequip or “SomnoFlo” from Kent Scientific)
Elizabethan collars (Rat from Kent Scientific, cat. no. EC404VL‐10)
Medical blue pad
Gauze
Safety wash bottle
1‐ml syringes (e.g., from BD)
27G × 0.5 in. (13 mm) sterile needles (e.g., “PrecisionGlide” from BD)
Heating pad (e.g., RightTemp from Kent Scientific)
Laser speckle contrast imager (e.g., PeriCam imaging system from Perimed)
Acquisition software (e.g., PIMsoft from Perimed)

#### Placement

1 Follow Basic Protocol 1 for the nymphal tick exposure and place an Elizabethan collar around guinea pigs’ necks with ticks on their heads.
  Avoid placing the nymphal ticks too close together. For the laser speckle contrast image acquisition, there should be at least 3 to 4 mm between nymphal ticks to make it easier to assess each tick's impact on the skin. Otherwise, cumulative effects from multiple ticks may be observed.

#### Depilation of guinea pigs

For effective laser speckle contrast image acquisition, bare skin needs to be exposed to the camera of the laser speckle contrast imaging system. Thus, hair removal is required from furry animals. Due to the tick placement, the fur at the tick bite site is already trimmed down to a stubble. Application of a depilatory cream allows removal of the remaining stubble to expose bare skin.

2 At a desired time‐point post tick placement, anesthetize guinea pigs with isoflurane for 2 to 3 min in an induction chamber.
  Use 4% isoflurane at 2 L/min flow rate with a conventional isoflurane vaporizer and at 1 L/min flow rate with a SomnoFlo.
3 Remove the Elizabethan collar from the anesthetized guinea pig, if used.
4 Place the guinea pig on a blue pad and place a nose cone over the guinea pig's snout to maintain isoflurane anesthesia.
5 Apply a generous layer of depilatory cream to the tick bite site.
  a. It is recommended to do the depilation after tick placement. The hair stubble is essential for the tick placement protocol. Depilation before tick placement significantly reduces the attachment success.
  b. Incubation time depends on the animal model:
    i. Guinea pigs require at least 3 to 4 min for optimal hair removal.
    ii. Mice require 20 to 30 s and should be monitored carefully.
  c. Do not leave the depilatory cream on beyond the optimal time to avoid chemical burns and killing of the nymphal tick. These burns may appear 24 to 48 hr after depilation, so continue to monitor them.
6 After the incubation time has elapsed, remove the depilatory cream quickly, but thoroughly using wet gauze or running water. Be careful not to dislodge attached ticks.
  a. Wipe the excess cream by wiping gently with the grain of the hair (from the front toward the back). This will minimize accidental dislodgement of attached ticks.
  b. With guinea pigs, you can remove residual cream with water from a safety wash bottle or under a running tap, if available. For the former, place the guinea pig on its side and rub gently over the depilated area with a soft sponge or your gloved finger while squirting water from the safety wash bottle on the area. For the latter, place the guinea pig on your lower arm and hand with the guinea pig's mandible resting on the tips of your index and middle fingers. Ensure that the guinea pig's snout is not submerged in the running water stream, or your guinea pig will drown. For mice, a safety wash bottle can be used to rinse off residual depilatory cream. Make sure that all cream is removed completely, as remaining residue can cause chemical burns and scabbing.
7 Gently dab the animal with some tissue paper to dry it off. Ensure that the animal is fully dry before returning it to the cage.
8 Replace the Elizabethan collar on the guinea pig, if used.
9 Return the guinea pigs to their triple‐containment cages (if ticks remain attached) or their home cage for recovery from the anesthesia.
10 Continue to monitor the guinea pigs until fully recovered from anesthesia.

#### Laser speckle contrast image acquisition

Laser speckle contrast imaging acquisition requires that the animal is lying as still as possible. We recommend general anesthesia with ketamine/xylazine, as it also slows guinea pig breathing, but other general anesthesia protocols may work as well. We do not recommend the use of isoflurane, because nose cones may interfere with image acquisition on the head. Also, breathing should be as shallow as possible because the animal moves under the camera while breathing. This matters because laser speckle contrast imaging records the animal over a preset period (typically 30 to 60 s) rather than just taking a single image. The more the animal moves due to breathing under the camera, the more noise is generated in the acquired data.

11 Weigh guinea pigs to determine anesthesia volume per guinea pig.
12 Anesthetize guinea pigs by intraperitoneal injection of a Ketamine (50 mg/kg) and Xylazine (5 mg/kg) mixture (see Reagents and Solutions).
13 Place the anesthetized animal on a heating pad at 37°C, apply lubricant eye ointment, and place it underneath the laser speckle contrast imaging camera.
  a. We use a 1 × 1–cm (1 cm^2^) area for acquisition.
  b. We acquire 16 images/s for 1 min.
  c. The resolution should be as high as possible. We operate at 0.02 mm resolution as the highest we can get on our system.
  d. Our system is set to a working distance of 10 cm. The camera distance is manually adjusted for each animal and each field of view.
14 Using the supplier's acquisition software (in our case PIMsoft from Perimed), record the animal for 1 min.
15 Move the animal to the next field of view by sliding the heating pad around on the bench. Avoid moving the camera for every field of view change.
16 Once all ticks have been imaged, either return the animal to the triple‐containment cage for recovery or process it further as required.

#### Laser speckle contrast image processing

**Figure 5:**
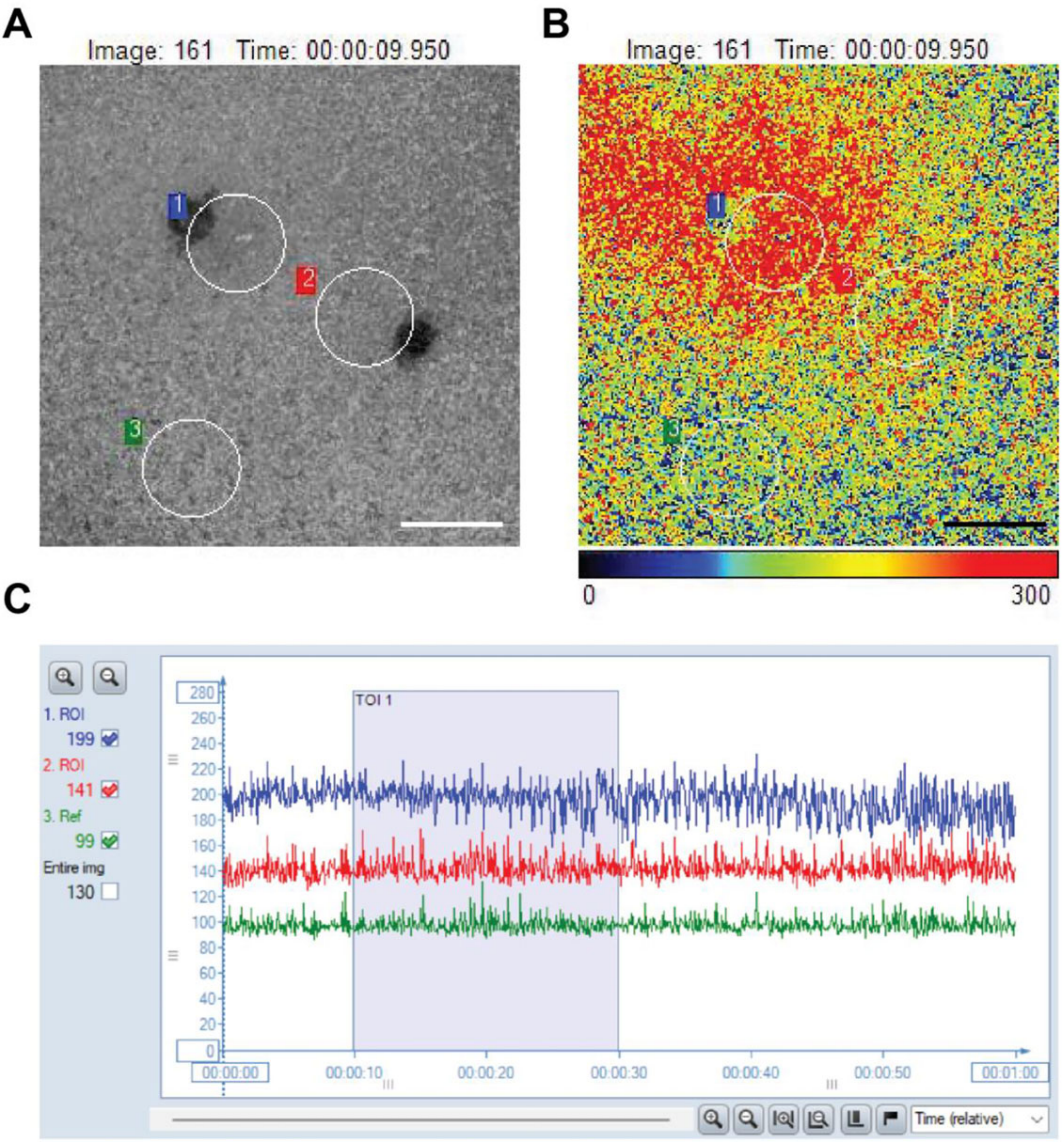
Example output image of data acquired with a Perimed laser speckle contrast imager. (**A**) Grey scale image of 1‐cm^2^ imaging area on a guinea pig showing two ticks. White circles numbered 1 to 3 are the regions of interest (ROI) measured for data acquisition. (**B**) Laser speckle measurements of tick‐bite sites on a tick‐sensitized guinea pig. Perfusion images. Scale: Blue (0) = no signal, red (300) = high signal. Scale bar, 2 mm. (**C**) Recording profile for the 3 ROIs. These examples are all drawn from the supplier's software, PIMsoft V.1.5 (Perimed).

17 Using the supplier's acquisition software (in our case PIMsoft from Perimed), draw a region of interest (ROI) for each attached tick in the image (Fig. 5). Place the ROI mask close to the tick, which should capture any inflammatory signal at the tick bite site.
  a. We recommend excluding the tick in the ROI. The tick obscures the animal's skin and therefore blocks out true signal from the cellular infiltrate.
  b. Copy/paste the ROI from the first tick for all the other ticks.

18 For each image, also draw a ROI away from all the ticks as a reference site. This will serve as a background reading,
19 Per image, subtract the value of the reference site from all the values from the tick bite sites to get signal proportion above the background (Δ perfusion units). This will normalize values between images, which makes them comparable between images.
20 Collate all the Δ perfusion units by groups and present them as grouped scatter plots.
  For the data analysis, it can be crucial to consider guinea pigs as a source of data variability, particularly when multiple ticks were collected from each guinea pig. That needs to be acknowledged as a random factor in the statistical analysis.

## REAGENTS AND SOLUTIONS

### Anesthesia

Anesthesia for guinea pigs was prepared as a Ketamine (50 mg/kg) and Xylazine (5 mg/kg) mixture (“Zetamine” Ketamine stock from VetOne and “Rompun” Xylazine stock from Dechra). The amount of each anesthetic required for the general anesthesia is calculated as follows:

Ketamine:

$$x=\frac{50\;mg\cdot kg^{-1}\times GP\;weight\;in\;grams}{anesthetic\;concentration\;in\;mg\cdot ml^{-1}}$$

Xylazine:

$$x=\frac{5\;mg\cdot kg^{-1}\times GP\;weight\;in\;grams}{anesthetic\;concentration\;in\;mg\cdot ml^{-1}}$$

For example, using a 100 mg/ml Ketamine stock solution and a 20 mg/ml Xylazine stock solution to anesthetize a 400 g heavy guinea pig, we require:

$$\frac{50\;mg\cdot kg^{-1}\times \;400\;g}{100\;mg\cdot ml^{-1}}=200\;\mu l\;Ketamine\;stock$$

$$\frac{5\;mg\cdot kg^{-1}\times \;400\;g}{20\;mg\cdot ml^{-1}}=100\;\mu l\;Xylazine\;stock$$

for a total intraperitoneal injectable volume of 300 µl. Undiluted Ketamine/Xylazine mixtures are not stable at room temperature for storage. We therefore recommend to set up the anesthesia fresh on the day before or on the day of the procedure.

## COMMENTARY

### Notes on Protocol Efficacy

#### Tick placement (Basic Protocol 2)

Tick attachment success is highly dependent on breaking diapause in ticks by sufficient sunlight exposure before placement. With our tick placement protocol, we observed average tick attachment of 91.6% (*A. americanum*), 88.1% (*I. ricinus*), 90.9% (*I. scapularis*), and 97.5% (*R. sanguineus*) for 60 guinea pigs for each tick species, respectively. The rate of attachment failure was low. Only in 8.3% (*A. americanum*), 15% (*I. ricinus*), 11.3% (*I. scapularis*), and 1.7% (*R. sanguineus*) of exposed guinea pigs did we observe a failure of >5 nymphal ticks to attach (Fig. 2). The exception was *D. variabilis* that had a lower attachment success with only 59% of ticks attaching. More than 5 nymphal ticks failed to attach in 83.3% of guinea pigs, which meant that an excess number of ticks needed to be placed on the animals. *D. variabilis* consistently showed a much slower drive to bite and attach. They also showed a clear preference for the base of the ears, the ears, and around the eyes to bite; presumably, because the guinea pig skin is thinner in these locations, making it easier for *D. variabilis* to draw blood with its short hypostome.

#### Recording analysis app (Basic Protocol 4)

The deep learning neural network underlying the recording analysis app significantly cuts down the analysis time of the behavioral recordings from several weeks to a few hours. However, despite substantial training, the identification of scratch bouts is variable and not without fail. The average of correctly assigned events is ∼77.8% (±12.5%) per recording, with a range from 35.2% to 95.4%. Since the algorithm was trained mostly on recordings of sensitized guinea pigs, the correct assignment of scratch bout is more accurate for highly sensitized guinea pigs than for tick‐naive unexposed and first‐time exposed guinea pigs (Fig. 4). This makes it a requirement to manually assess all events that were collected by the app as scratch events to the tick placement site.

### Critical Parameters

#### Tick placement (Basic Protocol 1)

As stated in Basic Protocol 1, the exposure of nymphal ticks to sunlight prior to tick placement to break diapause is crucial to the success of the protocol. Insufficient light exposure prevents the nymphs from biting. Another aspect to consider is the age of the nymphs. If they only recently molted or they are too old, they may be uninterested in feeding. The fur stubble is also crucial for nymphal tick attachment. Full depilation of skin reduces the likelihood of tick biting as the ticks’ dorsal sensilla are not stimulated.

#### Tick imaging (Basic Protocol 3)

It is recommended to acquire images of all ticks from the same collection with the same magnification. This will allow direct visual comparison of ticks on images, making them useful for presentation and publication. For the generation of the scutal index, this may be irrelevant as the scutal index is produced per image and is scale independent. It is critical, however, that scutum width is measured at the widest point of the scutum for all ticks and that the alloscutum length is measured as centrally as possible from the scutum to the alloscutum's rear. If not, the research may artificially inflate or deflate the scutal index for tick artificially.

#### Scratch bout quantification (Basic Protocol 4)

As previously stated, guinea pigs freeze when they perceive a threat. This includes any humans they can see, or sudden loud noises they hear. The freezing impulse is so strong that it suppresses scratching and will affect scratch bout quantification.

#### Laser speckle contrast imaging (Basic Protocol 5)

The distance of the camera from the area to be imaged is critical for the quality of the data. Our model requires a fixed distance of 10 cm from the imaged object. The greater the departure from that distance in either direction, the lower the accuracy of the signal that is detected. Ignoring the optimum distance value can generate significant artifacts in the data. Considering that the animal must be breathing for image acquisition, there will be noise in the data due to the body motion that comes with steady breathing. This is observable in the sinus curve of the acquired data in the software. The greater the amplitude of the sinus curve, the greater the motion in the animal due to breathing. Therefore, it is essential to record the animal over a designated time period. We recommend 60 s. This way, the user can choose a 20‐s section for the averaged data analysis, excluding any area of excessive sinus curve amplitude or of sudden increase/drop in signal detection.

### Troubleshooting

There are common challenges associated with these protocols. In Table 1, we are offering solutions to the most commonly observed issues a researcher may encounter when applying these protocols.

**Table 1:** Troubleshooting Guide for Tick Placement, Python Code Use, and Laser Speckle Contrast Imaging

Problem	Possible cause	Solution
*Basic Protocol* *1*		
Majority of nymphal ticks do not bite and move away	Not enough sunlight exposure to break diapause	Extend period of sunlight exposure
Majority of nymphal ticks are sluggish and do not bite	Nymphal tick may be too old	Replace with a new batch of nymphal ticks
*Basic Protocol* *4*		
Python script did not execute	The Python environment was not properly established in the bash environment	Establish and call your Python environment before running the scripts
	The directories were not correct	Check that there are no spelling mistakes in the directory's address
	The directories contained unescaped white space	Use the backslash “\” to escape white spaces as indicated in the protocol steps
*Basic Protocol* *5*		
Saturated signal across the whole skin	Detection scale upper limit set too low	Manually adjust upper detection limit in the software
	Residual depilatory cream caused by chemical burns of the skin	Rinse the depilated skin thoroughly with water

### Statistical Analysis

#### Assessing tick removal

When using Basic Protocol 2 for the assessment of tick removal, the tick removal data should ideally be collected as time‐to‐event data, also known as survival data, even when tick counting occurs at fixed time points. Time‐to‐event data notes the time post tick placement when a tick was found to be removed and placed at “1” in the data column. Should a tick still be attached at the end of data collection, then a “0” is placed in the data column with the time of the final time point in the time column. Note that time‐to‐event (survival) data can be analyzed in GraphPad Prism, but the available tests are limited. There are several functions that can analyze time‐to‐event data in GraphPad Prism, which are automatically applied in the Results section. For two groups, the Log Rank (Mantel–Cox) test can be used when the outcome matters most. The Gehan–Breslow–Wilcoxon test is an option if early events are frequent and carry particular weight in the study. For a first analysis, these modalities can offer insights into one's data. Other software, like the freely available R, offers more flexibility and more advanced options for data analysis. We recommend going this route for publication purposes. Package “survival” and “survminer” contain all the necessary functions. For example, they offer the more appropriate Peto–Peto modification of the Gehan–Breslow–Wilcoxon test, which is less conservative. For data with >2 groups, the “survminer” package also contains a function called “pairwise_survdiff”, for pairwise comparison with integrated *p*‐value correction. They also offer the Cox proportional hazard model. The advantage of the Cox proportional hazard model is that it can account for data hierarchy and produces odds ratios. Note that if you have >2 groups to analyze, you need to perform a pairwise post hoc analysis to find where the statistically significant difference is, while correcting your *p*‐values for multiple comparisons. The “lsmeans” function from the “emmeans” package can be employed to easily facilitate ad hoc pairwise comparison with integrated *p*‐value correction. In general, we recommend consulting a statistician before undertaking the experiment and for data analysis.

#### Analyzing scutal index data

We recommend plotting scutal index data collected according to Basic Protocol 3 as grouped scatter plots. The choice of data analysis is dependent on your experimental design. If ticks were collected from the same guinea pig at multiple time points, the data analysis requires data pairing in the analysis. If multiple ticks were collected from the same guinea pig, then a hierarchical analysis may be required. Mixed regression models can address data pairing and the inclusion of guinea pigs as a random factor (data hierarchy). Therefore, it is recommended to collect ticks from each guinea pig into their own tube. The assessment of data hierarchy usually requires the use of the interclass correlation coefficient (ICC) derived from regression models. You can also statistically compare the output of a linear mixed model that uses guinea pigs as a random factor to the output of a standard linear model. If statistical significance is observed, then the random factor needs to be included in the data analysis, increasing its complexity. These types of analyses are best conducted in more sophisticated software like R or SAS. In general, we recommend consulting a statistician before undertaking the experiment and for data analysis.

#### Analyzing scratch bout data

Determining the appropriate statistical analysis for the scratch bout data depends on the experimental design. Since scratch bout data is count data, there is the option to analyze the data by quasi‐Poisson or negative binomial regression. This has the advantage that guinea pigs can be integrated as a random effect in the data analysis. Further, it must be understood that the scratch bout analysis rarely renders zero values if the recording is long enough, because guinea pigs also scratch for routine grooming purposes, which is indistinguishable from scratches directed toward ticks due to sensitization. Thus, it must be assessed if zero‐truncation has a significant impact on the analysis. In general, one would run all model types and use, e.g., the Akaike Information Criterion to determine which model is the most appropriate for your data. In general, we recommend consulting a statistician before undertaking the experiment and for data analysis.

#### Analyzing Δ perfusion units’ data

Determining the appropriate statistical analysis for the Δ perfusion units’ data depends on the experimental design. If data is only collected once from each guinea pig, then a one‐way statistical test, like a *t*‐test (2 groups), ANOVA (>2 groups), or their non‐parametric counterparts may suffice. However, if each guinea pig is imaged more than once, a two‐way mixed model may be a more appropriate way to analyze the data. The latter also has the benefit that guinea pigs can be included as a random effect in the data analysis. In general, we recommend consulting a statistician before undertaking the experiment and for data analysis.

### Understanding Results

#### Tick removal data (Basic Protocol 2)

**Figure 6:**
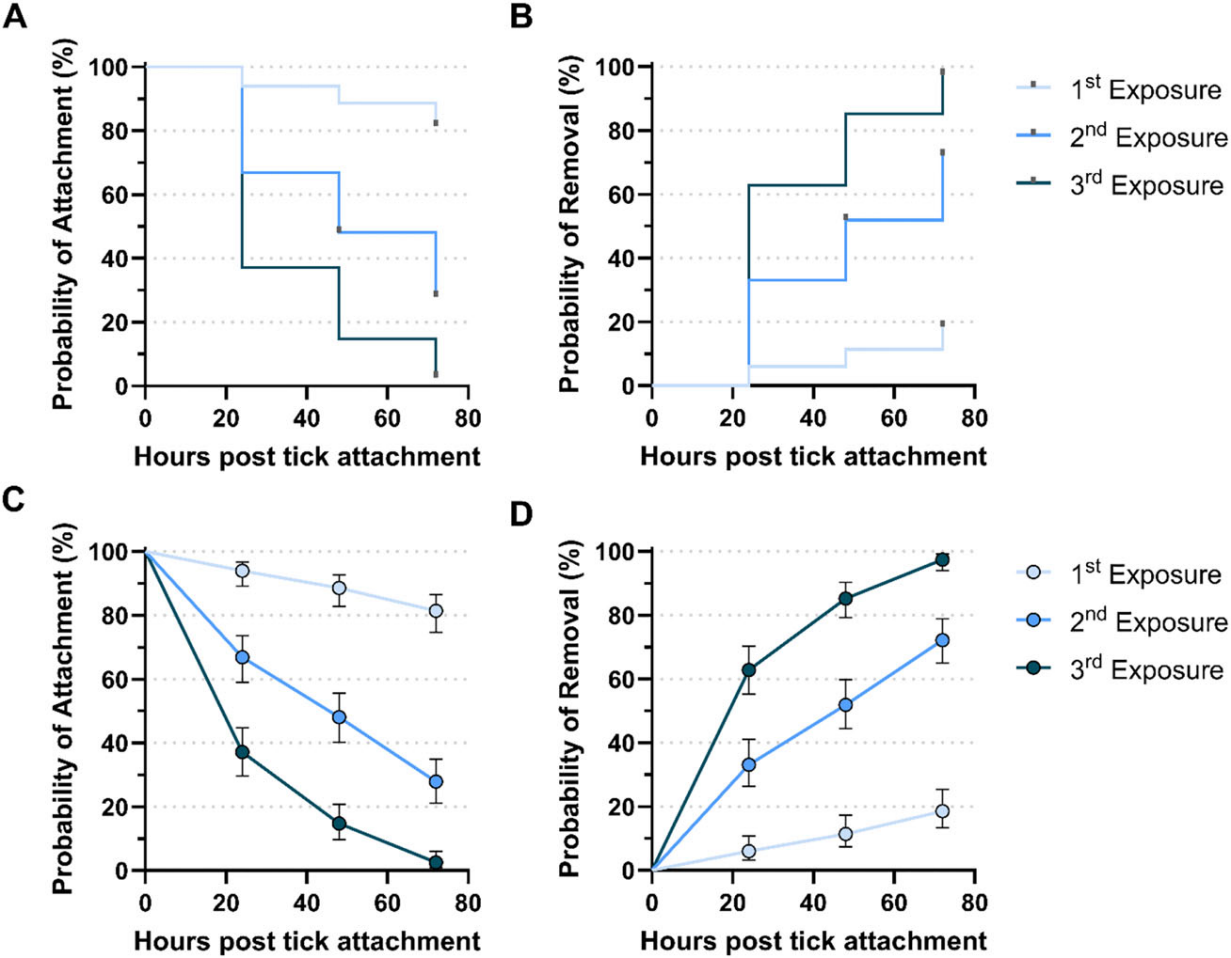
(**A**‐**D**) All graphs are different versions of a Kaplan–Meier plot showing the same data. Per graph, we show three weekly spaced consecutive tick exposures of Hartley guinea pigs (GPs) exposed to *I. scapularis* (*N* = 11 GPs; *N* = 483 ticks). Animals are tick‐naive for the 1^st^ exposure and tick‐sensitized for subsequent exposures. (**A**) Standard Kaplan–Meyer plots showing probability of tick attachment (%). (**B**) Standard inverted Kaplan–Meyer plots showing probability of tick removal (%). (**C**) Smoothed Kaplan–Meyer plots showing probability of tick attachment (%). (**D**) Smoothed, inverted Kaplan–Meyer plots showing probability of tick removal (%).

Time‐to‐event data are best plotted as a Kaplan–Meier graph (Fig. 6). Kaplan–Meier graphs can be inverted depending on whether you wish to show probability of attachment or probability of removal at a given time point (Fig. 6A‐B). Further, Kaplan–Meier graphs for data collected at fixed time points can be smoothed to resemble line graphs rather than the typical step plot (Fig. 6C‐D). Successful induction of itch‐induced tick removal is observed by increased tick removal success with each subsequent tick exposure. Failure to acquire itch‐induced tick removal is observed when there is a meaningful increase in tick removal with each subsequent tick exposure.

#### Scutal index data (Basic Protocol 3)

**Figure 7:**
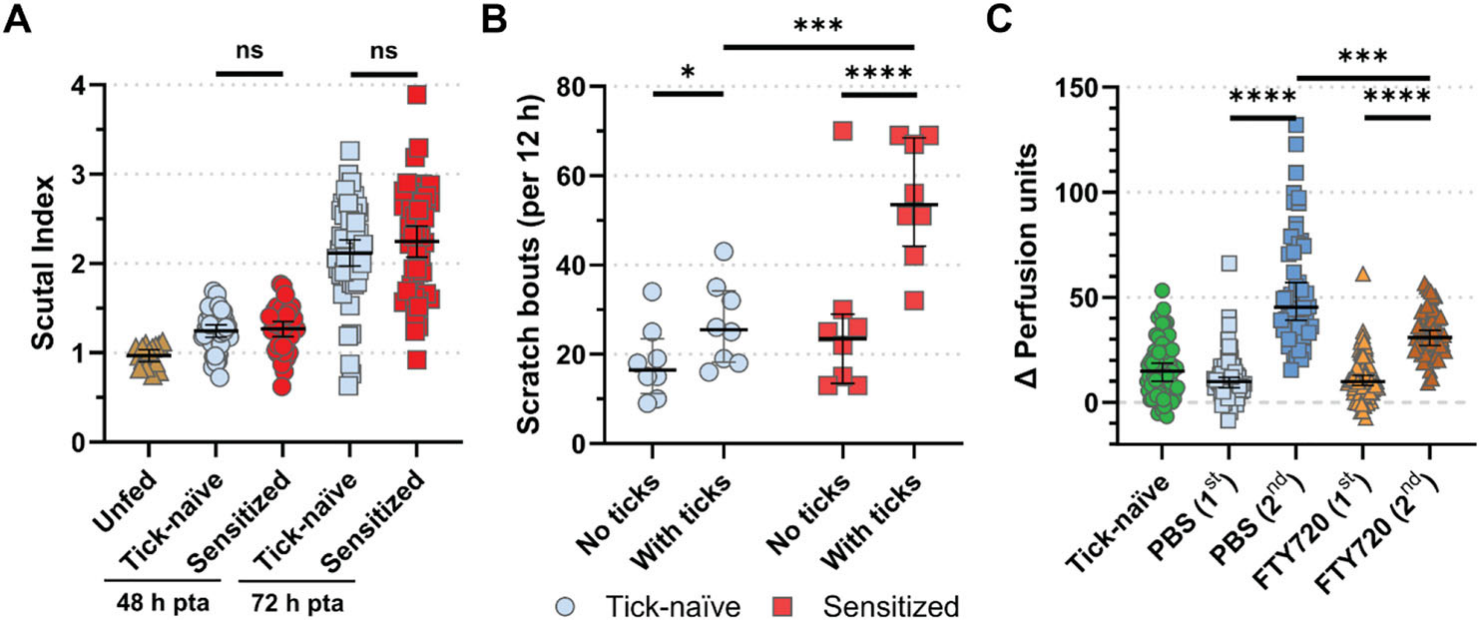
(**A**) Scatter plot: Scutal index values of nymphal ticks fed on tick‐naive (48 hr pta, *N* = 42; 72 hr pta, *N* = 54) and sensitized (48 hr pta, *N* = 38; 72 hr pta, *N* = 49) guinea pigs (GPs), or unfed (*N* = 16). Two‐way robust ANOVA (excluding unfed): sensitization state (tick‐naive vs tick‐sensitized; *p* = .3229); time (48 hr pta vs 72 hr pta; *p* < .0001). Mean ± 95% CI shown. (**B**) Scratch bouts per 12 hr in tick‐naive and tick‐sensitized GPs (*N* = 8/group). The same GPs were analyzed first without, then with ticks attached (total *N* = 181 and 169 ticks, respectively). Pairwise estimated marginal means: tick‐naive without and with ticks (*p* = .0231); tick‐sensitized without and with ticks (*p* < .0001); tick‐naive vs tick‐sensitized guinea pigs both with ticks (*p* < .0001). Median ± IQR shown. (**C**) Scatter plot: Delta Perfusion units (tick‐bite site signal – image background signal). Tick‐naive, PBS (first tick exposure; *N* = 73); PBS (second tick exposure; *N* = 48); FTY720 (first tick exposure; *N* = 72); FTY720 (second tick exposure; *N* = 62). Pairwise estimated marginal means: PBS (first vs second tick exposure; *p* < .0001); FTY720 (first vs second tick exposure; *p* < .0001); second tick exposure (FTY720 vs PBS; *p* = .0006. Median ± IQR shown. Figure legends and figures are from Doehl et al. (2026).

Scutal index data is best presented as grouped scatter plots (Fig. 7A). Whether you should use mean or median as the central tendency marker (black horizontal line for each data set) depends on whether your data is normally distributed or not. For error bars, we recommend the use of 95% confidence intervals when the mean is used, and interquartile ranges when the median is used. However, you may choose differently depending on what you wish to show with your error bars. The data example shows that blood feeding of ticks is not synchronous. Some ticks may start feeding sooner than others, while female nymphs will consume more blood than male nymphs.

#### Scratch bout data (Basic Protocol 4)

Scratch bout data is count data (discrete data) as their data values are always whole numbers (integers). This means that scratch bout data is not on a continuous scale, and should not be analyzed with parametric tests, which assume that your data is on a continuous scale (see above). Data presentation can be done as a grouped scatter plot using median and interquartile ranges for central tendency and error bars, respectively (Fig. 7B). This data presentation makes it easy to spot meaningful differences in scratch bouts between groups.

#### Δ perfusion units’ data (Basic Protocol 5)

Δ perfusion units' data is best presented as grouped scatter plots (Fig. 7C). This presentation makes it easy to spot differences between groups. Whether you should use mean or median as the central tendency marker (black horizontal line for each data set) depends on whether your data is normally distributed or not. For error bars, we recommend the use of 95% confidence intervals when the mean is used, and interquartile ranges when the median is used. In our experience, Δ perfusion units’ data is usually heavily positively skewed, particularly for sensitized guinea pigs. Note that perfusion units are an arbitrary scale, and that Δ perfusion units are merely the difference between the signal at the tick bite site and the image background (see above).

### Time Considerations

#### Basic Protocol 1

Including preparation of triple‐containment cages, this protocol takes ∼5 hr to complete with 6 guinea pigs. Upscaling is possible, but we do not recommend handling >9 guinea pigs between two people. Note that there is usually a good 60 min of free time between guinea pig recovery from ketamine/xylazine anesthesia and checking tick attachment. This interval will be shorter with 9 guinea pigs. Triple‐containment cage setup can also be completed the day before, which will save time on the day of tick exposure.

#### Basic Protocol 2

The duration of this protocol is fully dependent on the chosen time points on which you wish to assess tick removal. Note that the same time considerations must be made as for Basic Protocol 1 on the day of the tick exposure. If 72 hr post tick placement is your last time point, then this protocol will take 4 days in total.

#### Basic Protocol 3

The duration of this protocol is fully dependent on the chosen time points on which you wish to collect ticks from the guinea pigs. Note that the same time considerations must be made as for Basic Protocol 1 on the day of the tick exposure. Time commitment for tick collection and imaging can be several hours, depending on how many ticks are being collected. Since isoflurane anesthesia should be used on guinea pigs for tick collection, you may need to consider 10 min per guinea pig handled. The image collection can take some time, too. We recommend dedicating at least half of your working day to tick collection and imaging to produce quality data.

#### Basic Protocol 4

Note that the same time considerations must be made as for Basic Protocol 1 on the day of the tick exposure. The data acquisition usually occurs overnight, which only requires some time to set up the recording system and to collect the guinea pigs from the phenotyper cages the next day. If using a computer cluster, the uploading time of the recordings is dependent on your server connection. The analysis with the app only requires short intermittent attention from the researcher to call the next step. The running time is dependent on available computing power and the number of recordings being analyzed in a session. Visual confirmation of scratch bout assignment to short recording clips is the most time‐consuming step and depends on how many recordings were analyzed and how many scratch bouts were detected. But this last step can take a couple of days to complete but is not time sensitive.

#### Basic Protocol 5

Note that the same time considerations must be made as for Basic Protocol 1 on the day of the tick exposure. The time commitment on the day of the laser speckle contrast imaging is moderate and largely dependent on the number of animals being imaged. With a recording length of 1 min per region of interest and an average of 5 regions of interest per guinea pig, each guinea pig is usually processed in ∼10 min, excluding induction of anesthesia, which can be done for the next guinea pig prior to imaging the guinea pig anesthetized before. Overall, 90 min may be required to image 6 guinea pigs, including setting up the laser speckle imager.

### Author Contributions

**Ronja A. Frigard**: Methodology; writing—original draft; writing—review and editing. **Serena Doh**: Data curation; methodology. **Charles S. Grugan**: Data curation; methodology. **Yanling Liu**: Conceptualization; software; writing—original draft; writing—review and editing. **Sara I. Mirbagheri**: Visualization; writing—review and editing. **Luana A. Rogerio**: Investigation; methodology; validation; visualization; formal analysis. **Eva Iniguez**: Investigation; writing—review and editing. **Tiago D. Serafim**: Investigation; writing—review and editing. **Aline da Silva Moreira**: Investigation; writing—review and editing. **Jack Collins**: Supervision. **Daniel E. Sonenshine**: Conceptualization; data curation; methodology; writing—original draft; writing—review and editing. **Jesus G. Valenzuela**: Conceptualization; funding acquisition; methodology; project administration; supervision; writing—original draft; writing—review and editing. **Johannes S. P. Doehl**: Conceptualization; data curation, formal analysis; methodology; validation; visualization; writing—original draft; writing—review and editing.

### Conflict of Interest

The authors declare no conflicts of interest.

## Data Availability

The data that support the findings of this study are available from the corresponding author upon reasonable request. The underlying scripts for the recording analysis app are available for download from GitHub at: https://github.com/IDSS‐NIAID/tick‐scratch‐detection.

## References

[cpz170477-bib-0001] Anderson, J. M. , Moore, I. N. , Nagata, B. M. , Ribeiro, J. M. C. , Valenzuela, J. G. , & Sonenshine, D. E. (2017). Ticks, ixodes scapularis, feed repeatedly on white‐footed mice despite strong inflammatory response: An expanding paradigm for understanding tick‐host interactions. Frontiers in Immunology, 8, 1784. 10.3389/fimmu.2017.01784 PMC5741700 29326693

[cpz170477-bib-0002] Beard, C. B. , Eisen, L. , & Eisen, R. J. (2021). The rise of ticks and tickborne diseases in the united states‐introduction. Journal of Medical Entomology, 58(4), 1487–1489. 10.1093/jme/tjab064 PMC9620473 33939806

[cpz170477-bib-0003] Brites‐Neto, J. , Duarte, K. M. , & Martins, T. F. (2015). Tick‐borne infections in human and animal population worldwide. Veterinary World, 8(3), 301–315. 10.14202/vetworld.2015.301-315 PMC4774835 27047089

[cpz170477-bib-0004] Doehl, J. S. P. , Serafim, T. D. , Doh, S. , Grugan, C. S. , Iniguez, E. , Rogerio, L. , Frigard, R. , Dey, R. , Cecilio, P. , Gu, X. , Tseng, P.‐Y. , Moreira, A. D. S. , Minai, M. , Oristian, J. , Ackerman, H. , Brooks, S. , Percopo, C. , Ng, S.‐P. , Alves, D. A. , … Valenzuela, J. G. (2026). Discovery of an adaptive neuroimmune response driving itch and fast tick removal with implications for preventing pathogen transmission. Advanced Science, 13(32), e17742. 10.1002/advs.202517742 PMC13252611 41589694

[cpz170477-bib-0005] Eisen, L. (2018). Pathogen transmission in relation to duration of attachment by Ixodes scapularis ticks. Ticks and Tick‐borne Diseases, 9(3), 535–542. 10.1016/j.ttbdis.2018.01.002 PMC5857464 29398603

[cpz170477-bib-0006] Eisen, R. J. , & Paddock, C. D. (2021). Tick and tickborne pathogen surveillance as a public health tool in the United States. Journal of Medical Entomology, 58(4), 1490–1502. 10.1093/jme/tjaa087 PMC8905548 32440679

[cpz170477-bib-0007] Erickson, S. , & Kim, B. S. (2019). Research techniques made simple: Itch measurement in clinical trials. Journal of Investigative Dermatology, 139(2), 264–269.e1. 10.1016/j.jid.2018.12.004 PMC8922716 30665580

[cpz170477-bib-0008] Felix, R. , & Shuster, S. (1975). A new method for the measurement of itch and the response to treatment. British Journal of Dermatology, 93(3), 303–312. 10.1111/j.1365-2133.1975.tb06496.x 1191538

[cpz170477-bib-0009] Gebbia, J. A. , Bosler, E. M. , Evans, R. D. , & Schneider, E. M. (1995). Acquired resistance in dogs to repeated infestation with Ixodes scapularis (Acari: Ixodidae) reduces tick viability and reproductive success. Experimental and Applied Acarology, 19(10), 593–605. 10.1007/BF00048814 8556959

[cpz170477-bib-0010] Heeman, W. , Steenbergen, W. , van Dam, G. , & Boerma, E. C. (2019). Clinical applications of laser speckle contrast imaging: A review. Journal of Biomedical Optics, 24(08), 1–11. 10.1117/1.JBO.24.8.080901 PMC6983474 31385481

[cpz170477-bib-0011] Huercha , Song, R. , Li, M. , Fan, X. , Hu, Z. , Wu, L. , Li, Y. , Zhang, W. , Zhang, Y. , Ma, Y. , & Bayin, C. (2020). Characterization of glutathione S‐transferase of Dermacentor marginatus and effect of the recombinant antigen as a potential anti‐tick vaccine. Veterinary Parasitology, 279, 109043. 10.1016/j.vetpar.2020.109043 32070900

[cpz170477-bib-0012] Kryda, K. , Naito, M. , Fujii, T. , Hodge, A. , & Maeder, S. (2025). Treatment and control of Haemaphysalis longicornis infestations on dogs using a formulation of sarolaner, moxidectin and pyrantel (Simparica Trio(R)). Parasites & Vectors, 18(1), 117. 10.1186/s13071-025-06747-6 PMC11948799 40140872

[cpz170477-bib-0013] Lee, K. N. , Pellom, S. T. , Oliver, E. , & Chirwa, S. (2014). Characterization of the guinea pig animal model and subsequent comparison of the behavioral effects of selective dopaminergic drugs and methamphetamine. Synapse, 68(5), 221–233. 10.1002/syn.21731 PMC3980732 24436154

[cpz170477-bib-0014] Madison‐Antenucci, S. , Kramer, L. D. , Gebhardt, L. L. , & Kauffman, E. (2020). Emerging tick‐borne diseases. Clinical Microbiology Reviews, 33(2), e00083‐00018. 10.1128/CMR.00083-18 PMC6941843 31896541

[cpz170477-bib-0015] Meiners, T. , Hammer, B. , Göbel, U. B. , & Kahl, O. (2006). Determining the tick scutal index allows assessment of tick feeding duration and estimation of infection risk with Borrelia burgdorferi sensu lato in a person bitten by an Ixodes ricinus nymph. International Journal of Medical Microbiology, 296 Suppl 40, 103–107. 10.1016/j.ijmm.2006.01.048 16524770

[cpz170477-bib-0016] Narasimhan, S. , Cibichakravarthy, B. , Wu, M.‐J. , Holter, M. M. , Walsh, C. A. , & Goodrich, J. A. (2024). Laboratory Management of Mammalian Hosts for Ixodes scapularis‐Host‐Pathogen Interaction Studies. Comparative Medicine, 74(4), 235–245. 10.30802/AALAS-CM-24-036 PMC11373684 39289828

[cpz170477-bib-0017] Nicholson, W. L. , Sonenshine, D. E. , Noden, B. H. , & Brown, R. N. (2019). Ticks (Ixodida). In G. R. Mullen & L. A. Durden (Eds.), Medical and Veterinary Entomology (3rd ed., pp. 603–672). Academic Press. 10.1016/B978-0-12-814043-7.00027-3

[cpz170477-bib-0018] Otaki, H. , Sonobe, J. , Murphy, M. , Cavalleri, D. , Seewald, W. , Drake, J. , & Nanchen, S. (2018). Laboratory evaluation of the efficacy of lotilaner (Credelio) against Haemaphysalis longicornis infestations of dogs. Parasites & Vectors, 11(1), 448. 10.1186/s13071-018-3032-0 PMC6090816 30071885

[cpz170477-bib-0019] Owen, J. P. , Gibbs, A. , Jones, C. R. , Brunner, J. L. , Mason, K. , Noh, S. M. , & Scoles, G. A. (2025). Linked empirical studies reveal the cumulative impact of acquired tick resistance across the tick life cycle. Ticks and Tick‐borne Diseases, 16(3), 102460. 10.1016/j.ttbdis.2025.102460 40112618

[cpz170477-bib-0020] Sonenshine, D. E. , & Roe, R. M. (2013). Biology of Ticks ( D. E. Sonenshine & R. M. Roe , Eds. 2nd ed., Vol. 2). Oxford University Press.

